# Impact of revised antral follicle count threshold on PCOS diagnosis: A comparative clinical study

**DOI:** 10.1371/journal.pone.0358554

**Published:** 2026-10-15

**Authors:** Saloni Kamboj, Neena Malhotra, Reeta Mahey, Taruna Arora, Aditi Goyal, Snigdha Soni, Anshu Jyoti, Spondita Banerjee, Rintu Kutum

**Affiliations:** 1 Department of Obstetrics and Gynaecology, All India Institute of Medical Sciences, New Delhi, Delhi, India; 2 Division of Reproductive Biology, Maternal and Child Health, Indian Council of Medical Research, New Delhi, Delhi, India; 3 Department of Computer Science, Ashoka University, Sonipat, Haryana, India; UFRN: Universidade Federal do Rio Grande do Norte, BRAZIL

## Abstract

**Objective:**

To evaluate the clinical impact of the revised antral follicle count (AFC) threshold on polycystic ovary syndrome (PCOS) diagnosis.

**Methods:**

A retrospective cross-sectional observational study including women diagnosed using the Rotterdam criteria, stratified by AFC: 12–19 (low AFC) and ≥20 (high AFC). Clinical, metabolic, and hormonal parameters were compared against controls and amongst low and high AFC group.

**Results:**

Of 336 PCOS women, 208 (62%) had an AFC between 12 and 19 and would not meet the revised ≥20 follicle threshold for the diagnosis of polycystic morphology. These women had worse metabolic and androgenic profiles than controls, and were similar to the high AFC group.

**Conclusion:**

Excluding low AFC women may misclassify patients who require clinical care.

## Introduction

Polycystic ovary syndrome (PCOS) is one of the most common endocrine–metabolic disorders affecting reproductive-aged women. It is characterized by ovarian dysfunction, hyperandrogenism (HA), and frequently insulin resistance, and represents a leading cause of anovulatory infertility.

Diagnostic criteria for PCOS have evolved over time.The 1990 NIH criteria [1] required the presence of both chronic anovulation and clinical and/or biochemical hyperandrogenism, after exclusion of other etiologies, without incorporating ovarian morphology. In 2003, the Rotterdam consensus (ESHRE/ASRM-sponsored workshop) [2] expanded the definition to include polycystic ovarian morphology (PCOM) as a third diagnostic feature. According to the Rotterdam criteria, PCOS is diagnosed when any two of the following three features are present: (i) oligo/anovulation (OA), (ii) hyperandrogenism (HA), and (iii) polycystic ovaries on ultrasonography. This broadened definition resulted in four phenotypes: A (OA + HA + PCO), B (OA + HA), C (HA + PCO), and D (OA + PCO).

Under the original Rotterdam definition, PCOM was defined as ≥12 antral follicles measuring 2–9 mm in diameter in at least one ovary and/or ovarian volume >10 mL. However, advances in ultrasound technology over the past two decades have substantially improved follicle detection. As a result, the 2018 International Evidence-Based Guideline for PCOS [3] recommended increasing the follicle number threshold to ≥20 follicles per ovary when high-resolution transvaginal probes (≥8 MHz) are used, with the aim of improving diagnostic specificity and reducing overdiagnosis.

Polycystic ovarian morphology remains one of the most frequently observed components of PCOS. A recent multicentric study [4] demonstrated PCOS phenotype C (HA/PCO) as the most prevalent type (40.8%) followed by phenotypes D (OA/PCO) (24.6%), indicating ahigh prevalence PCOM, particularly where ovarian morphology plays a defining role.Another prospective study showed 88.7% of PCOS patients having polycystic ovaries [5]. Kim et al. [6] demonstrated a mean AFC of 15–16 in their multicentric study on PCOS patients.

Revising the AFC threshold may therefore substantially alter diagnostic classification, especially among women presenting with irregular menstruation or hyperandrogenism and an intermediate follicle count (12–19). Such reclassification could have implications for clinical management, metabolic risk assessment, and reproductive counseling.

The objective of this study was to evaluate the impact of the revised AFC threshold (≥20) on the diagnostic classification of adult women previously diagnosed with PCOS using the Rotterdam criteria. We further compared the clinical and biochemical characteristics of women excluded under the new threshold with both control subjects and those fulfilling the revised criteria. Additionally, we also aimed to evaluate the diagnostic performance and concordance of anti-Müllerian hormone **(**AMH) in identifying PCOS under both the traditional (AFC ≥ 12) and revised (AFC ≥ 20) morphological definitions.

Lastly, we sought to identify the classification of women presenting with irregular menstruation (IM) or hyperandrogenism (HA) and an AFC ranging from 12 to 19.

## Materials and methods

### Subjects

This retrospective cross-sectional observational study was conducted at the Gynaecology Outpatient Department (OPD) of the All India Institute of Medical Sciences (AIIMS), New Delhi. The study included women evaluated over a five-year period up to 2022 who were screened for PCOS as part of routine clinical care. Relevant clinical, biochemical, and ultrasonographic data were extracted from medical records for analysis. A total of 335 women were recruited as cases and 163 women as controls. All of them were in the age ranging between 20 and 40 years. Cases were categorized as PCOS in accordance with the Rotterdam criteria and women fulfilling the criteria were recruited in our study. Oligo-anovulation included oligomenorrhea and/or anovulation. Oligomenorrhea is characterized by having fewer than eight menstrual cycles per year or cycles exceeding 35 days in length. Amenorrhea, on the other hand, referred to the absence of menstruation for over three months, provided that pregnancy was not the cause. Hyperandrogenism was defined as either clinical, assessed by the presence of hirsutism and/or acne, or biochemical, determined by elevated serum total testosterone levels.Clinical hyperandrogenism was assessed using the modified Ferriman–Gallwey (mFG) score. Hirsutism was defined as an mFG score ≥8. Acne was recorded based on clinical examination and considered supportive of hyperandrogenism in conjunction with other features.Women who were on any oral contraceptives were asked to interrupt the medications for at least 3 months before they could be enrolled in the study. Any women who had consumed insulin sensitizers or lipid lowering agents were also excluded from the study. Transvaginal ultrasound examinations were performed with a high frequency probe (9.0 MHz, Voluson, S-6, GE Healthcare, USA) by trained personnel. The evaluation was performed in the early follicular phase of the cycle or periods of amenorrhoea. All the visible follicles were visualized and manually counted concurrently, in three planes, measuring between 2–8 mm in each ovary, and the total AFC was calculated by totalling. Patient information was recorded in an Electronic Medical Record System (Microsoft Excel). Controls were defined as women with regular menstrual cycles, no clinical or biochemical hyperandrogenism, and no polycystic ovarian morphology on ultrasound (AFC < 12 follicles per ovary and ovarian volume ≤10 mL). Additionally, women with known endocrine or metabolic disorders, including thyroid dysfunction, diabetes mellitus, hyperprolactinemia, congenital adrenal hyperplasia, Cushing’s syndrome, or androgen-secreting tumors, were excluded. Relevant clinical evaluation and laboratory testing were performed to exclude these conditions.

The study was approved by the ethics committee of the Institute (IEC-491/07.10.2016) and all participants gave informed written consents to be part of the study.

### Clinical and biochemical measurements

All subjects had their Blood pressure (BP) measured. Anthropometric measurements, such as body weight, height, waist circumference (WC) were noted. For the calculation of BMI, weight (kg) was divided by height squared (m2). In all subjects, samples for fasting lipid profile consisting of triglycerides (TG), high-density lipoprotein (HDL) cholesterol and low-density lipoprotein (LDL) Cholesterol, fasting glucose, insulin, HbA1c, and 2-h 75 g glucose tolerance testwere drawn and evaluated in the laboratory.Testosterone measurements were available for all PCOS cases and a subset of controls (20%) due to clinical workflow constraints. Analyses involving biochemical hyperandrogenism were restricted to subjects with available data.Serum levels of total T, free T and sex hormone-binding globulin were measured using a radioimmunoassay (Siemens, Los Angeles, CA, USA). The intra and inter-assay coefficients of variation were 4.0–11.0% and 5.9–12.0% for total T, respectively, and 4.0–17% and 8.0–18.3% for free T, respectively. In women with PCOS, serum LH, FSH, estradiol and anti-Mullerian hormone (AMH) were measured.AMH was quantified using the Beckman Coulter AMH Gen II ELISA assay (Beckman Coulter Inc., Brea, CA, USA).

### Statistical analysis

Data was analysed and the mean±standard deviation or the median value with an interquartile range was derived upon. For data showing a Gaussian distribution geometric means and 95% confidence intervals were used for expression. Student’s t-test or analysis of variance were used to compare continuous parameters whereas chi-square test was applied to categorical data.

All data analyses were performed using Statistical Package for the Social Sciences software (version 23.0, IBM SPSS, NY, USA), and statistical significance was set at a two-sided P-value of <0.05.When required, a single transformation (natural logarithmic or square root) was applied uniformly across the entire dataset for a given variable to improve normality. Parametric tests (Student’s t-test or one-way analysis of variance [ANOVA]) were then performed on the transformed scale, and results were expressed as geometric means with 95% confidence intervals where appropriate. When normality assumptions were not met or could not be achieved, non-parametric tests (Kruskal–Wallis rank-sum test) were used, followed by Dunn’s post-hoc test for pairwise comparisons.Categorical variables are presented as frequencies and percentages and were compared using Pearson’s chi-squared test or Fisher’s exact test, as appropriate.

Receiver operating characteristic (ROC) curves were plotted to evaluate the diagnostic performance of anti-Müllerian hormone (AMH) in discriminating PCOS and controls under two AFC thresholds (≥12 and ≥20). The area under the curve (AUC), sensitivity, specificity, and optimal cutoff points were derived using the Youden index. Cohen’s Kappa statistic assessed agreement between AMH-based classification and AFC-based diagnosis.

Missing data were handled using complete-case analysis. For variables not available in all participants (e.g., testosterone levels in controls), analyses were restricted to subjects with available data. No statistical imputation was performed.

Additional analyses, including clustering and visualization, were conducted in R Statistical Programming Language (version 4.4.1).

### Unsupervised machine learning

Unsupervised machine learning was performed to investigate the potential clusters based on the clinical parameters only. We removed the group information and performed unsupervised clustering on the clinical data of subjects without NA values (Total = 476 out of 497, Low AFC = 192, High AFC = 122, Control = 162) using the random forest algorithm to compute the similarity across subjects due to the mixed clinical parameters (categorical and numerical variables). The similarity among subjects were estimated using a random forest algorithm with 50,000 decision trees in an unsupervised setting. The similarity matrix from the random forest algorithm was converted into a dissimilarity matrix (1 – similarity matrix), and Partitioning Around Medoids (PAM) was applied by varying cluster number (k) from 2 to 20. Based on average silhouette width, the best cluster number (k) was selected. Visualisation of the clustering was performed using a multidimensional scaling plot (MDS plot). The analysis was performed in R Statistical Programming Language (version R 4.4.1) and implementation of the algorithms and visualization were performed using randomForest package (version, 4.7.1.2), cluster package (version 2.1.8.1), ggplot2 package (version 3.5.2) and ggsci package (version 3.2.0).

## Results

Antral Follicle Count was measured for all the subjects with PCOS. Those having AFC of 12–19 were categorised as ‘low AFC’ (n = 208)

Comparison Of Clinical Characteristics Among the Low AFC Group, High AFC Group and Controls (Table 1)

**Table 1:** Clinical and anthropometric characteristics of women in the low AFC (12–19), high AFC (≥20), and control groups.

	Low AFC Group (n = 208)	High AFC group (n = 127)	Controls (n = 163)	*P* – Value	*P*-Value(1,2)	*P*-Value(2,3)	*P*-Value(1,3)
**Anthropometric Parameters**							
**Age (Years)**	30.30 ± 3.8	29.50 ± 3.44	30.93 ± 6.12	0.034	0.376	0.577	0.028
**BMI (kg/m**^**2**^)	25.43 ± 3.84	25.82 ± 4.27	24.38 ± 3.87	0.004	1.000	0.034	0.007
**WC (cm)**	91.69 ± 9.72	90.68 ± 10.14	76.50 ± 11.37	<0.001	1.000	<0.001	<0.001
**Hirsutism**	99 (47.37)	73 (57.48)	4 (2.47)	<0.001			
**SBP (mmHg)**	120.44 ± 6.27	120.66 ± 6.59	126.33 ± 10.97	<0.001	1.000	<0.001	<0.001
**DBP (mmHg)**	79.78 ± 7.26	79.74 ± 6.72	68.83 ± 7.97	<0.001	1.000	<0.001	<0.001
**Cycles per year**	5.89 ± 3.81	5.74 ± 3.23	11.32 ± 4.11	<0.001	0.79	<0.001	<0.001
**mFG Score**	11.20 ± 3.81	11.40 ± 3.60	3.91 ± 2.79	<0.001	0.84	<0.001	<0.001

Continuous variables are presented as mean ± SD or median (IQR), as appropriate, and were compared using one-way analysis of variance (ANOVA) or Kruskal–Wallis test, followed by pairwise comparisons. Categorical variables are presented as n (%) and were compared using Pearson’s chi-squared test.

Comparison between controls and the low AFC group (AFC 12–19), who would not satisfy the revised ≥20 follicle criterion for PCOM, revealed several noteworthy differences

BMI and waist circumference were significantly higher in the low AFC group than controls. Lab parameters also followed the same trail. Serum AMH and Total Testosterone were also significantly raised in the low AFC group. Corresponding trends were observed in LDL, fasting glucose, fasting Insulin and 75 g 2 hr Insulin and sugar levels thus indicating worse metabolic profiles in the low AFC group as compared to the controls. TG levels were similar in controls and low AFC group. The high AFC and low AFC group had no significant differences in terms of anthropometric measurements and laboratory values, thus indicating similar metabolic profiles. (Table 2, 3).

**Table 2:** Comparison of hormonal parameters among low AFC (12–19), high AFC (≥20), and control groups.

	Low AFC Group (n = 209)	High AFC group (n = 127)	Controls (n = 162)	*P* – Value	*P*-Value(1,2)	*P*-Value(2,3)	*P*-Value(1,3))
**Hormonal Parameters**							
**Testosterone (ng/dl)**	0.32 (0.1-1.0)	0.308 (0.044-1.0)	0.2405 (0.034-0.9)	<0.001	1.00	<0.001	<0.001
**LH (mIU/ml)**	5.9 (1.3-25.1)	5.4 (0.27-24.1)	6.205 (0.315-48.4)	0.247	0.731	0.612	0.141
**FSH (mIU/ml)**	5.52 ± 1.61	5.58 ± 1.48	8.21 ± 7.50	<0.001	1.000	<0.001	<0.001
**AMH (ng/ml)**	10.06 ± 3.68	11.03 ± 4.02	5.24 ± 4.66	<0.001	0.112	<0.001	<0.001

Values are presented as mean ± standard deviation or median (range), as appropriate. Pairwise comparisons were conducted between low AFC vs high AFC (P1), low AFC vs controls (P2), and high AFC vs controls (P3). AFC: antral follicle count; LH: luteinizing hormone; FSH: follicle-stimulating hormone; AMH: anti-Müllerian hormone.

**Table 3:** Comparison of metabolic parameters among low AFC (12–19), high AFC (≥20), and control groups.

	Low AFC Group (n = 209)	High AFC group (n = 127)	Controls (n = 162)	*P* – Value	*P*-Value(1,2)	*P*-Value(2,3)	*P*-Value(1,3))
**Metabolic Parameters**							
**TG (mg/dl)**	116.5 (36 −444.7)	116.1 (35- 444.9)	120 (37-320)	0.8572	1.00	1.00	0.871
**LDL (mg/dl)**	100.38 ± 26.38	105.26 ± 27.98	90.87 ± 31.10	<0.001	0.383	0.004	<0.001
**HDL (mg/dl)**	42.33 ± 9.47	42.08 ± 8.60	48.45 ± 10.55	<0.001	1.000	<0.001	<0.001
**Fasting Glucose (mg/dl)**	85.41 ± 8.23	86.41 ± 7.64	89.83 ± 17.16	0.0014	1.000	0.001	0.044
**Fasting Insulin (Uu/ml)**	11.8 (3.65-72.6)	11.8 (1.26-72.6)	8.185 (0.22-81.5)	<0.001	0.761	<0.001	<0.001
**75 g OGTT 2hr Insulin (Uu/ml)**	77 (13.5-357)	70.4 (10.4-357)	16.23 (1.45-91.6)	<0.001	0.314	<0.001	<0.001
**75 g OGTT 2 hr Glucose (mg/dl)**	113.17 ± 28.66	115.86 ± 24.79	97.96 ± 23.80	<0.001	1.000	<0.001	<0.001

Values are presented as mean ± standard deviation or median (range), as appropriate. Pairwise comparisons were performed between low AFC vs high AFC (P1), low AFC vs controls (P2), and high AFC vs controls (P3). TG: triglycerides; LDL: low-density lipoprotein cholesterol; HDL: high-density lipoprotein cholesterol; OGTT: oral glucose tolerance test.

Increasing the AFC threshold for polycystic ovarian morphology from ≥12 to ≥20 follicles resulted in loss of the PCOM criterion in 228 of 335 women (68.1%). Of these, 128 women originally classified as phenotype A continued to fulfil the oligo-anovulation and hyperandrogenism criteria and were reclassified as phenotype B, while 62 remained phenotype A. All 13 women with phenotype B remained unchanged. Among women with phenotype C, 72 lost the PCOM criterion and were reclassified as having isolated hyperandrogenism, while 32 remained phenotype C. All 28 women with phenotype D were reclassified as having isolated oligo-anovulation. Overall, 100 of 335 women (29.9%) no longer fulfilled the Rotterdam diagnostic criteria for PCOS after applying the AFC ≥ 20 threshold. (Table 4)

**Table 4:** Phenotype-wise reclassification of women with PCOS following revision of the antral follicle count threshold for polycystic ovarian morphology from ≥12 to ≥20 follicles.

Original phenotype using AFC ≥ 12	n (%)	Classification after applying AFC ≥ 20	n (%)
A: OA + HA + PCOM	190 (56.7)	A: OA + HA + PCOM	62 (18.5)
		B: OA + HA	128 (38.2)
B: OA + HA	13 (3.9)	B: OA + HA	13 (3.9)
C: HA + PCOM	104 (31.0)	C: HA + PCOM	32 (9.6)
		Hyperandrogenism only	72 (21.5)
D: OA + PCOM	28 (8.4)	Oligo-anovulation only	28 (8.4)
**No longer fulfilling the Rotterdam criteria**	—		**100 (29.9)**

Data are presented as n (%). Percentages were calculated using the total cohort of women diagnosed with PCOS using an antral follicle count (AFC) threshold of ≥12 follicles.

AFC: antral follicle count; PCOM: polycystic ovarian morphology; OA: oligo-anovulation; HA: hyperandrogenism; PCOS: polycystic ovary syndrome.

On ROC analysis, AMH demonstrated excellent discriminatory ability for PCOS defined by the traditional AFC ≥ 12 criterion (AUC = 0.8253; 95% CI 0.78–0.86) (Fig 1a), with an optimal cutoff of 6.88 ng/mL, yielding a sensitivity of 81.3% and specificity of 73.0%.Using the revised AFC ≥ 20 definition, diagnostic accuracy declined (AUC = 0.6841; 95% CI 0.63–0.73) (Fig 1b), and the Youden-optimal cutoff shifted lower to 5.6 ng/mL, achieving higher sensitivity (93.6%) but poor specificity (35.4%).Agreement between AMH ≥ 6.88 ng/mL and PCOS by the old AFC ≥ 12 criterion was moderate (κ = 0.53, p < 0.001), but fell markedly for the AFC ≥ 20 classification (κ = 0.18, p < 0.001).Concordance plots showed that under the ≥ 20 cutoff, 42.6% of women were AMH-positive but AFC-negative, reflecting substantial diagnostic discordance. Thus, while AMH ≥ 6.88 ng/mL strongly aligns with clinically defined PCOS, the ≥ 20 follicle threshold excludes many biochemically hyper-AMH women sharing identical metabolic risk profiles.

**Fig 1:**
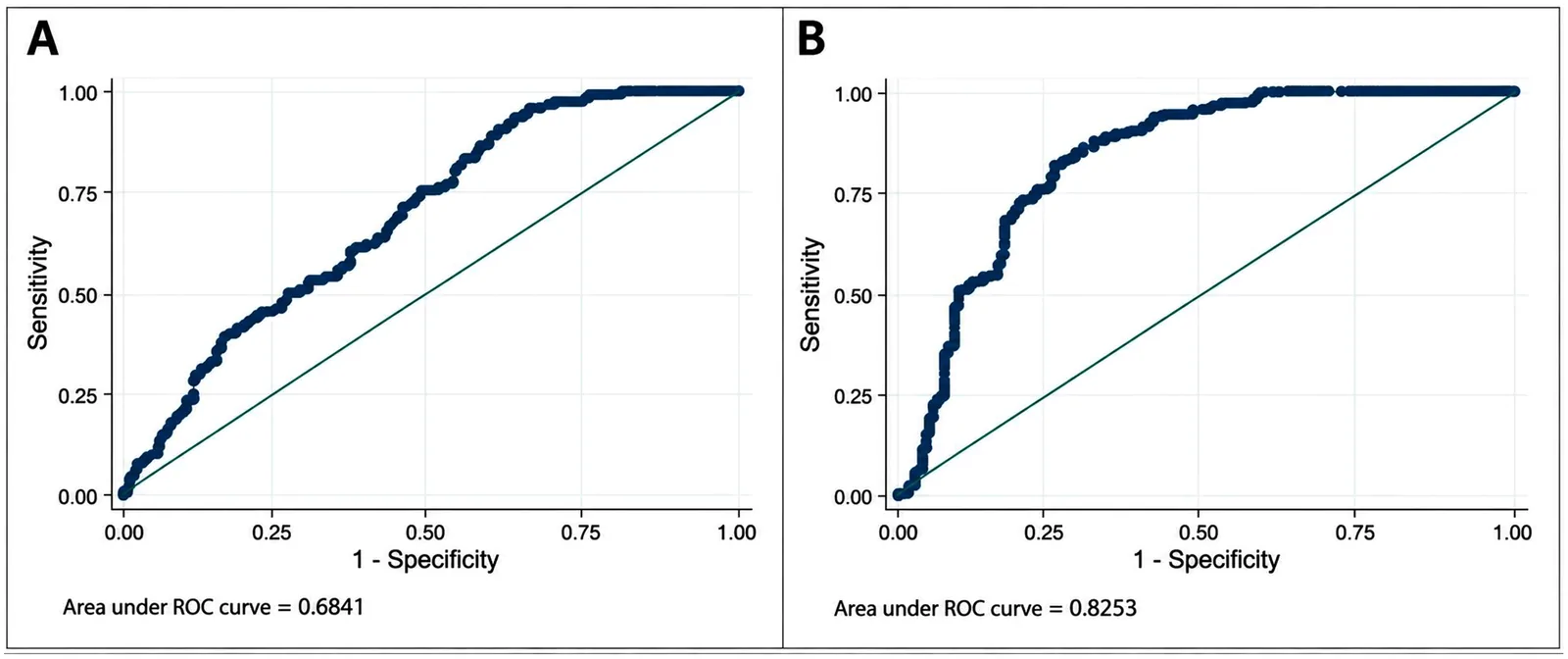
(a) ROC curve of AMH for PCOS diagnosis using AFC ≥ 12 criterion (AUC = 0.8253). **(b)** ROC curve of AMH for PCOS diagnosis using AFC ≥ 20 criterion (AUC = 0.6841).

### Unsupervised machine learning confirms no differences in low and high AFC groups

To explore potential latent grouping patternsin subjects with low and high AFC groups, we performed unsupervised clustering with the random forest machine learning algorithm and the partition around medoids algorithm. Based on the unsupervised clustering we observed two clusters (k = 2) with average silhouette width of 0.14 (Fig 2A). We observed that both Low AFC and High AFC subjects were in the Cluster 1, and 90.74% of control subjects were in Cluster 2 (147/162), and 9.26% (15/162) were in Cluster 1 (Fig 2B and 2C).

**Fig 2:**
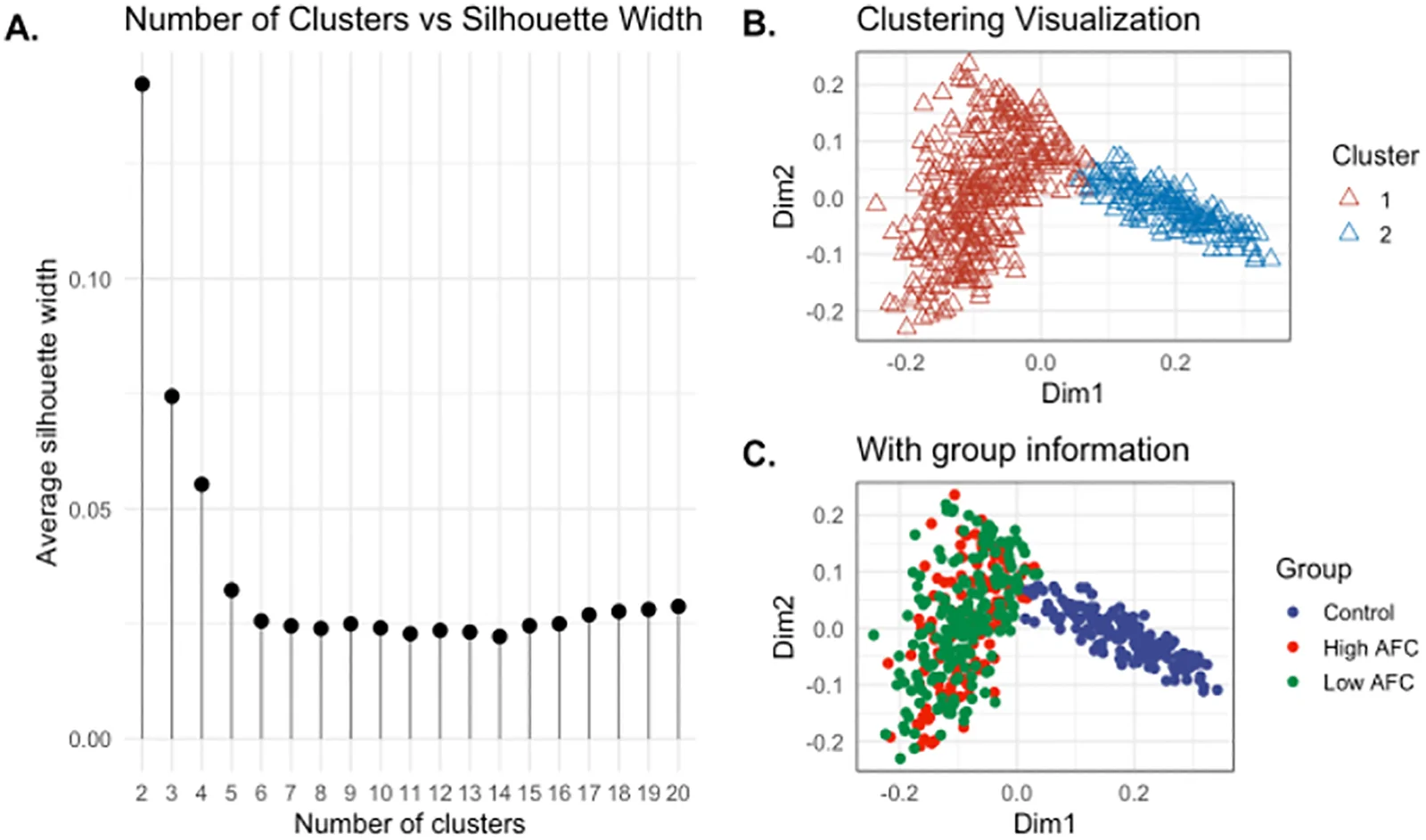
Unsupervised machine learning captures low and high AFC subjects into a single cluster group. A. Lollipop plot showing average silhouette width per cluster number (k) from 2 to 20; the higher width reflects the best cluster number (the best k is 2). B. Multidimensional scaling plot (MDS plot) represents the cluster memberships of the subjects. C. MDS plot based on the unsupervised clustering with the original group information of the subjects.

## Discussion

The advent of a new AFC cutoff for diagnosis of PCOS by the International Guideline Development Group, intrigued us whether it would be fair to those women who would thus be exempted from PCOS diagnosis. Rotterdam criteria considered women with AFC > 12 to be PCO, and therefore they needed either OA or HA to be diagnosed as PCOS and thus managed accordingly. On the contrary, patients with AFC between 12–19, who were earlier managed as PCOS, will be treated as normal as per the international guideline development group. We sought to understand the far-reaching implications of these changes through this study. Moreover, we tried to determine the position of these excluded women relative to the control group and the remaining patients.

Our findings demonstrate that increasing the AFC threshold from 12 to 20 would reclassify a substantial proportion (62.2%; 208/335) of women previously meeting PCOM criteria under Rotterdam. Importantly, this change does not uniformly eliminate PCOS diagnosis but predominantly affects phenotype classification, particularly in women in whom ovarian morphology was a defining feature (phenotypes C and D).Women fulfilling both oligo/anovulation and hyperandrogenism would remain diagnosed irrespective of follicle count. These findings highlight the sensitivity–specificity balance inherent in revising AFC thresholds and underscore the importance of evaluating clinical and endocrine context rather than relying solely on follicle number.This was reflected in the metabolic dysfunction evident in the low AFC group as reflected by the poorer metabolic parameters, such as BMI, lipid profiles (HDL and LDL profiles), and diabetic status along with greater androgen levels as compared to the controls. Interestingly, they were similar to the remaining PCOS patients in terms of anthropometric measurements, metabolic syndrome, and androgen levels. These findings point to the fact that a substantial proportion of women with PCOS visiting clinics for hyperandrogenism or irregular menstruation could be redefined as ‘not having PCOS’ under the new AFC cutoff despite requiring comparable management and clinical care.

Jonard in 2003 [7] cited the AFC threshold of 12 using a 7 MHz transducer with 99% specificityand 75% sensitivity. They had also compared clinical and hormonal parameters such as BMI, Serum testosterone, serum insulin and serum FSH and LH. All the parameters were significantly higher in PCOS patients compared to controls, as expected and as has been demonstrated in our study too.

Another study conducted by Chen et al. [8], diagnosed PCOS patients using NIH criteria. They used a transvaginal probe of 6 MHz and found the mean follicle number in PCOS patients as 10 with 85.2% sensitivity and 88% specificity.

A study was conducted across 5 different zones of India to find prevalence of PCOS and the phenotypic spectrum of PCOS [4]. Their results revealed that individuals with PCOS exhibited worse metabolic and hormonal profiles than the control group as has been evident in our study as well. Ethnicity seems to influence the expression of various PCOS phenotypes. They found PCOS phenotype C (HA/PCO) to be most prevalent (40.8%), followed by phenotype D (OA/PCO) (24.6%), A (HA/OA/PCO) (20.2%), and B (HA/OA) (14.3%). This highlights the need for region specific evidence as any change in AFC cutoff for PCO will have a greater impact on Indian women compared to Caucasian women. In our study, 62.2% of women were excluded from PCOS diagnosis as per new cutoff. When comparing the metabolic and hormonal milieu, they behaved similar to those with AFC > 20. In another Indian study, Kar et al [9] evaluated different PCOS phenotypes and suggested a cutoff of ≥12 follicles per ovary using 2D transvaginal ultrasonography and ≥10 follicles per ovary using 3D transvaginal ultrasonography among women diagnosed with PCOS.

A pilot study conducted in North Africa using 18-MHz transvaginal ultrasonography aimed to validate follicular and ovarian volume thresholds in women with PCOS [10]. They found LH to be 9.04 mIU/ml for PCOS group and 7 mIU/ml for the control group. Serum Testosterone levels were also found to be significantly higher in PCOS patients (0.779 ± 0.173 ng/ml) relative to the control group (0.416 ± 0.145 ng/ml), indicating hyperandrogenemia prevalent in 91.5% of PCOS patients versus 6.7% in the control group. The median estimated follicle count by the new and old ultrasound machines was 20 follicles and 12 follicles for patients, and 9 follicles and 6 follicles for the control group, respectively. The performance of the new ultrasound machine was evaluated against the old ultrasound machine using the ROC curve. For the diagnosis of ultrasound-based PCOS, the ROC curve analysis demonstrated a cutoff of 18 follicles with a sensitivity of 68.1% and a specificity of 100%. The AUC was 0.955 with a confidence interval of 0.914–0.995.

Anti Mullerian Hormone (AMH), though not yet used as a diagnostic criterion for PCOS, has been established as a surrogate marker for PCOS in various studies [11]. Dewaillyet al. [12] recommended the AMH threshold of 5 ng/ml to be 92% sensitive and 97% specific in PCOS patients. In another study in Indian PCOS women, serum AMH levels showed a positive correlation with anthropometric measurements and lipid profile parameters [13]. In our study, PCOS patients demonstrated significantly higher AMH levels compared to controls. Also, there was not much difference among the low and high AFC group for the same (10.06 ± 3.68 vs 11.03 ± 4.02 ng/ml)

ROC-based analysis supports AMH as a robust marker for PCOS within populations using the classical AFC ≥ 12 criterion, consistent with previous Indian and Asian data showing AUC 0.80–0.88 for AMH cutoffs around 6–7 ng/mL. However, when applying the newer AFC ≥ 20 threshold, AUC and agreement fall sharply (κ ≈ 0.18), suggesting AMH and AFC become desynchronized at higher follicle thresholds. This likely reflects the nonlinear relationship between small-antral follicle count and AMH secretion beyond the physiological saturation zone.By excluding women with intermediate follicle counts (12–19), many individuals with high AMH levels are reclassified as non-PCOS, thereby decreasing diagnostic concordance.Accordingly, our findings should be interpreted as evaluating the concordance of AMH with PCOS classification under revised AFC thresholds, rather than proposing AMH as a replacement for the complete Rotterdam diagnostic framework, which is in line with the recent recommendations for the diagnosis of PCOS.

From a practical standpoint, increasing the diagnostic threshold for polycystic ovarian morphology from ≥12 to ≥20 follicles would result in 29.9% of women no longer meeting the Rotterdam criteria for PCOS. Given that many of these women continued to demonstrate biochemical evidence of ovarian hyperactivity, including elevated AMH levels, these findings raise concerns that the revised threshold may reduce diagnostic sensitivity in symptomatic women with less marked follicular excess.

The observed discordance highlights the potential importance of considering ethnicity- and technology-specific factors when applying AFC thresholds. Variations in ovarian reserve markers across ethnic groups may reflect biological differences, environmental influences, or methodological variability in ultrasound resolution and follicle assessment. In our predominantly South Asian cohort, the impact of revising the AFC threshold may therefore differ from that observed in other populations. AMH, as a biochemical marker of follicular activity, demonstrated stable discriminatory performance and may serve as a supportive adjunct within the diagnostic framework. However, extrapolation of these findings to other ethnic groups should be undertaken cautiously. Future multicenter studies evaluating ethnicity- and technology-adjusted AFC and AMH thresholds may help refine diagnostic accuracy.Multivariable and unsupervised analyses further confirm that women with 12–19 follicles share similar metabolic and androgenic profiles to those with ≥20 follicles, emphasizing the limitations of a universal cutoff. Integrating AMH with hormonal and clinical parameters, rather than relying solely on numerical follicle thresholds, may yield a more inclusive and pathophysiologic valid diagnosis.

The unsupervised clustering analysis was exploratory and demonstrated limited separation, and therefore should not be interpreted as defining definitive phenotypic subtypes. Notably, 9.26% (15/162) of controls clustered with PCOS participants, suggesting that clinical and metabolic traits may exist along a continuum rather than within strictly dichotomous categories. However, given the cross-sectional design, the clinical significance of this observation cannot be determined, and longitudinal studies are needed to assess whether such individuals are at increased risk of developing PCOS or related metabolic abnormalities.

This study has several limitations. First, ultrasound examinations were performed in a routine clinical setting without formal blinding to patient history, and therefore measurement bias cannot be entirely excluded. Second, testosterone levels were not available for all control participants, which may have resulted in under-detection of biochemical hyperandrogenism within the control group. Third, the cross-sectional design precludes causal inference regarding long-term metabolic risk, disease progression, or treatment outcomes. Longitudinal studies are necessary to determine whether reclassification under revised AFC thresholds translates into meaningful differences in reproductive or cardiometabolic outcomes.

Even with all that has been discussed, we need to address the challenges faced by the low AFC group women which may be exempted from PCOS diagnosis. Rectifying their metabolic and hormonal imbalance and regular follow ups for any worsening of PCOS like symptoms sounds like a pragmatic approach.Although these women may not fulfill the revised morphological criterion, their endocrine and metabolic characteristics suggest that clinical surveillance and, where appropriate, lifestyle or pharmacologic interventions (e.g., insulin-sensitizing therapy) may still be warranted.

## Supporting information

S1 File PCOS Revised AFC Data. (XLSX)
