# Supplementary material for: Impact of revised antral follicle count threshold on PCOS diagnosis: A comparative clinical study
Source: PLoS One. 2026 Oct 15;21(10):e0358554. doi: 10.1371/journal.pone.0358554 (PMC13671520; doi:10.1371/journal.pone.0358554)
Supplement: Supplementary file 2 [file pone.0358554.s004.docx]

**RESPONSE TO REVIEWER**

**Reviewer #1**

**Reviewer Comment:**
*“Where do the AFC cutoffs of 12 (‘old’) and 20 originate and why is it important to study this? The authors assume familiarity. NIH criteria are also mentioned without explanation. Please improve the Introduction.”*

**Response**

We thank the reviewer for this insightful comment. We agree that the background regarding the evolution of AFC thresholds for polycystic ovarian morphology (PCOM) and the diagnostic criteria for PCOS was insufficiently detailed in the original submission.

To address this:

1. We have now clearly described:
   - The origin of the **AFC ≥12 threshold** from the 2003 Rotterdam consensus.
   - The rationale for revising this threshold to **AFC ≥20** in the 2023 International PCOS Guideline, primarily due to advancements in ultrasound resolution.
2. We have elaborated on:
   - The diagnostic frameworks of **NIH (1990)** and **Rotterdam (2003)** criteria.
   - How the inclusion of PCOM as a diagnostic criterion created phenotype heterogeneity.
3. We have clarified:
   - Why reassessing AFC thresholds is clinically relevant, especially in reproductive medicine populations where ovarian reserve markers directly influence stimulation strategies, prognosis, and risk stratification (e.g., OHSS).

These changes have been incorporated into the revised Introduction.

**Reviewer Comment:**
*“Does the Beckman Coulter AMH kit mean the Gen II? Please describe better, so that the results can be compared to previous studies. Also, remember to mention the assay used when comparing to previous studies.”*

**Response**

We thank the reviewer for highlighting this important methodological point. We agree that anti-Müllerian hormone (AMH) values are assay-dependent and that clear specification is essential for comparison with prior studies.

AMH was measured using the Beckman Coulter AMH Gen II ELISA assay (Beckman Coulter Inc., Brea, CA, USA). We have now explicitly specified the assay platform and manufacturer details in the Methods section.

These revisions have been incorporated in the Methods and Discussion.

**Reviewer Comment:**
How many of the low AFC group fulfill the volume > 10 mm2 criterion? Could the authors provide also the mean volumes?

Shall be providing data with mean ovarian volumes ( in th excel ).

**Reviewer Comment:**

*“The authors say 336 women would have missed the diagnosis if the AFC cutoff of 20 had been used. So, these 336 women would only have irregular cycles or hyperandrogenism? How about ovarian volume in these 336 women? What is the mean age of the women who would miss PCOS diagnosis?”*

**Response:**

We thank the reviewer for this important observation and agree that the wording in the Discussion may have overstated the impact of the revised AFC threshold.

The 335 women referred to represent those with an AFC between 12–19 (low AFC group) who fulfilled polycystic ovarian morphology (PCOM) under the Rotterdam definition (≥12 follicles) but would not meet the revised ≥20 follicle threshold. We have revised the manuscript to clarify that these women would no longer meet the **follicle-count criterion for PCOM**, rather than uniformly “missing” a PCOS diagnosis.

Importantly, not all women in this subgroup would lose the Rotterdam diagnosis. Women fulfilling both oligo/anovulation (OA) and hyperandrogenism (HA) would continue to meet diagnostic criteria irrespective of ovarian morphology. Loss of diagnosis would primarily apply to those in whom PCOM constituted one of the two required criteria (i.e., phenotypes C and D), particularly if the ovarian volume criterion (>10 mL) was not met. We have now explicitly clarified this in the revised Discussion.

The mean age of women in the AFC 12–19 group was **30.30 ± 3.8 years**, compared to **29.50 ± 3.44 years** in the AFC ≥20 group (p = 0.034), as shown in Table 1. This information has now been explicitly referenced in the Results section for clarity.

We have modified the Discussion to replace the phrase “would no longer meet PCOS diagnosis” with more precise language reflecting diagnostic reclassification rather than absolute loss of diagnosis.

**Reviewer Comment:**

*“How do the authors explain that the cutoff of AMH is higher for the lower AFC group than for the higher one?”*

**Response:**

We thank the reviewer for this insightful observation. The apparent paradox arises because the ROC-derived AMH cutoff is dependent on the reference standard used to define PCOS, rather than directly reflecting follicle number alone.

When PCOS was defined using the traditional AFC ≥12 criterion, the case group included a broader spectrum of ovarian morphology (AFC ≥12), and the ROC analysis identified 6.88 ng/mL as the optimal balance between sensitivity and specificity. However, when the more stringent AFC ≥20 threshold was applied, women with intermediate follicle counts (12–19), many of whom had elevated AMH, were reclassified as non-PCOS. As a result, the distribution of AMH values between the newly defined “PCOS” and “non-PCOS” groups overlapped substantially.

In this context, the ROC algorithm shifted the Youden-optimal cutoff downward to 5.6 ng/mL in an attempt to preserve sensitivity. However, this occurred at the cost of marked specificity reduction (35.4%), reflected in the decline in AUC (0.825 to 0.684) and kappa agreement (0.53 to 0.18). Thus, the lower AMH cutoff under the ≥20 definition reflects diagnostic discordance introduced by the revised AFC threshold.

**Reviewer Comment:**

*“Did the authors consider age when assessing AMH and AFC? Barbagallo et al. noted age-specific AMH cutoffs.”*

**Response:**

We thank the reviewer for highlighting the important influence of age on AMH and AFC. We acknowledge that both parameters decline with advancing age and that age-specific thresholds have been proposed, including by Barbagallo et al. (JCEM 2020), who demonstrated variation in optimal AMH cutoffs across age strata.

In our primary analysis, ROC curves were constructed using the entire cohort without age stratification. While the mean age differed slightly between the AFC 12–19 and ≥20 groups (30.30 ± 3.8 vs 29.50 ± 3.44 years; p = 0.034), the absolute difference was modest.

## There was no significance noted in age parameter between two AFC Groups.

## Reviewer Comment:

“Do the authors think the AFC cutoff should be ethnicity-dependent, like FG-scores are for clinical hyperandrogenism?”

## Response:

We thank the reviewer for this thoughtful and clinically relevant question. Ethnic variation in PCOS phenotypic expression is well recognized, particularly for clinical hyperandrogenism, where Ferriman–Gallwey (FG) score thresholds are ethnicity-specific due to differences in hair follicle sensitivity and androgen responsiveness.

Whether antral follicle count (AFC) thresholds should similarly be ethnicity-dependent is an important consideration. Ovarian reserve markers, including AFC and AMH, have been shown in some studies to vary across populations, potentially reflecting genetic, environmental, metabolic, and anthropometric influences. Additionally, differences in baseline ovarian morphology between ethnic groups have been reported.

However, unlike FG scoring, which measures a clinical manifestation influenced by peripheral androgen sensitivity, AFC reflects ovarian follicular dynamics and is measured using imaging technology that is subject to methodological variability (e.g., probe frequency, operator expertise, counting protocol). Thus, variability in AFC thresholds may reflect both biological and technical factors.

Our cohort consisted predominantly of South-east Asian women. While our findings suggest that the ≥20 follicle threshold may result in significant reclassification within this population, we cannot determine from this study whether optimal AFC cutoffs should be ethnicity-specific. Multicentre studies across diverse populations, using standardized ultrasound protocols, would be required to establish whether population-specific thresholds improve diagnostic precision.

We have added a paragraph in the Discussion acknowledging the potential influence of ethnicity on AFC and AMH thresholds and highlighting the need for population-based validation.

Reviewer #2

### **Reviewer Comment:**

“When talking about people, rounding to the closest whole number is preferable to stating 62.2%. An improved version might state: 208 (62%) of the 335 women had AFC between 12 and 19 and would be excluded under new guidelines.”

### **Response:**

We thank the reviewer for this helpful suggestion. We agree that when referring to individuals rather than purely statistical measures, reporting whole numbers alongside rounded percentages improves clarity and readability.

Accordingly, we have revised the Abstract to state:

“208 (62%) of the 335 women had an AFC between 12 and 19 and would not meet the revised ≥20 follicle threshold.”

We have also ensured consistent rounding of percentages throughout the manuscript where appropriate.

**Reviewer Comment:**

*“‘ovarian volume > 10 cm2.’ Most likely the authors meant: > 10 mL?”*

**Response:**

We thank the reviewer for identifying this typographical error. The correct unit is **ovarian volume >10 mL**, as per the Rotterdam criteria. This has now been corrected throughout the manuscript.

### Reviewer Comment:

"‘this AMENDMENT needs to be highlighted’ — Did the authors mean ‘this ITEM needs to be’?"

### Response:

We thank the reviewer for pointing out the imprecise wording. We intended to refer to the **revision of the antral follicle count (AFC) threshold in the updated PCOS guideline**. The term “amendment” has now been replaced with “revision of the follicle threshold” to improve clarity and precision.

### Reviewer Comment:

“The study period was July 2018 to June 2019. Why did it take 6½ years to submit?”

Response :

Study is a retrospective study from last 5 years from an institute ( utill 2022 ) ( Added in methodology )

### **Reviewer Comment:**

“Hyperandrogenism can be either clinical, assessed by hirsutism and/or acne, or clinical, which was measured by Total testosterone.”

### **Response:**

We thank the reviewer for identifying this typographical error. The term “clinical” was inadvertently repeated in the original sentence. We have corrected the text to clearly distinguish between clinical and biochemical hyperandrogenism.

The revised sentence now reads:

“Hyperandrogenism was defined as either clinical, assessed by hirsutism and/or acne, or biochemical, determined by elevated serum total testosterone levels.”

We have carefully reviewed the manuscript to ensure consistent terminology throughout.

**Reviewer Comment:**

*“(75-g oral glucose tolerance test) were WITHDRAWN” → should be “were drawn.”*

**Response:**

We thank the reviewer for identifying this typographical error. The term “withdrawn” has been corrected to “drawn” in the revised manuscript.

**Reviewer Comment:**

Results "Coming to the comparison between the controls and low AFC group, who have been exempted from PCOS as per the latest recommendations, we observed some noteworthy differences." ---> Comparing the controls to the low AFC group who would have been exempted from PCOS as per......

**Response:**

We thank the reviewer for this suggestion. The sentence has been revised for clarity and precision. It now reads:

“Comparison between controls and the low AFC group (AFC 12–19), who would not satisfy the revised ≥20 follicle criterion for PCOM, revealed several significant differences.”

**Reviewer Comment:**

*"Rotterdam criteria considered women with AFC > 12 to be PCO, and therefore they needed either OA or HA to be diagnosed as PCOS and thus managed accordingly." -----> Rotterdam criteria define PCOS as women with AFC > 12 accompanied by either OA or HA.”*

**Response:**

We thank the reviewer for highlighting the need for greater precision in describing the Rotterdam criteria. We have revised the sentence to clarify that PCOS is defined by the presence of any two of three features: oligo/anovulation, hyperandrogenism, or polycystic ovarian morphology, rather than requiring ovarian morphology in all cases. The text has been corrected accordingly.

### Reviewer Comment:

### "On the contrary, patients with AFC between 12-19, who were earlier managed as PCOS, will be treated as normal as per the international guideline development group." ------> According to the new AFC cut off of 19, patients with AFC between 12-19, who were earlier managed as PCO, will be treated as normal.....

### Response:

We thank the reviewer for this suggestion. We have revised the sentence to more accurately reflect the diagnostic framework. The updated text now clarifies that women with AFC 12–19 would no longer meet the revised ≥20 follicle threshold for PCOM and may be reclassified depending on the presence of other Rotterdam criteria, rather than being universally considered “normal.”

### **Reviewer Comment:**

“‘Jonard in 2003’ — Add number of the Jonard citation (11).”

### **Response:**

We thank the reviewer for this suggestion. The citation has now been appropriately referenced as Jonard et al. (2003) [11] in the revised manuscript.

### **Reviewer Comment:**

“‘Another study conducted by Chen et al.,’ — Add number of the citation of Chen (12).”

### **Response:**

We thank the reviewer for pointing this out. The citation number has now been added, and the text has been revised to read:

“Another study conducted by Chen et al. [12] …”

The reference list has been cross-checked to ensure consistency throughout the manuscript.

### **Reviewer Comment:**

“A study was conducted across 5 different zones of India to find prevalence of PCOS and the phenotypic spectrum of PCOS.” — Add citation number.

### **Response:**

We thank the reviewer for this suggestion. The appropriate citation number has now been added to the sentence in the revised manuscript.

The sentence now reads:

“A study conducted across five different zones of India to determine the prevalence and phenotypic spectrum of PCOS [X]…”

### **Reviewer Comment:**

### "In another Indian prospective study byKar et al on various types of phenotypes suggested a cut-off of 12 and 10 follicle numbers per ovary ---> add citation (5) and add AFC: In another Indian prospective study by Kar et. al. (5) on various types of phenotypes suggested a cut-off of 12 AFC and 10 follicles per ovary in

### **Response:**

We thank the reviewer for this suggestion. The sentence has been revised for clarity and precision, and the appropriate citation has been added. The text now reads:

“In another Indian prospective study, Kar et al. [5] evaluated different PCOS phenotypes and suggested a cutoff of ≥12 follicles per ovary using 2D transvaginal ultrasonography and ≥10 follicles per ovary using 3D transvaginal ultrasonography among women diagnosed with PCOS.”

We believe this revision improves clarity and accurately reflects the findings of the cited study.

### Reviewer Comment:

### "A pilot study done in the North African continent aimed to validate follicular and ovarian thresholds by an 18-MHz ultrasound imaging in polycystic ovary syndrome.-------> Needs citation= (15)---> A pilot study done in the North African continent aimed to validate follicular and ovarian thresholds by an 18-MHz ultrasound imaging in polycystic ovary syndrome found......(15 ).

### Response:

We thank the reviewer for this suggestion. The appropriate citation [15] has now been added to the sentence. The paragraph has also been revised for clarity and grammatical accuracy. The updated text now reads:

“A pilot study conducted in North Africa using 18-MHz transvaginal ultrasonography aimed to validate follicular and ovarian volume thresholds in women with PCOS [15]…”

Reviewer #3

**Reviewer Comment:**

*“Data availability does not fully meet PLOS’s ‘without restriction’ standard. I suggest making the data available in a public repository.”*

**Response:**

We thank the reviewer for this important suggestion. In accordance with PLOS data-sharing policies, we have now deposited the fully de-identified dataset in a publicly accessible repository. The repository link and DOI have been added to the Data Availability Statement.

All personal identifiers were removed prior to upload to ensure participant confidentiality.

### **Reviewer Comment:**

“The references are not marked in the text.”

### **Response:**

We thank the reviewer for pointing this out. The in-text citations have now been carefully inserted and cross-checked throughout the manuscript in accordance with the journal’s referencing style. We have ensured that all statements supported by literature are appropriately referenced and that citation numbering corresponds accurately with the reference list.

### **Reviewer Comment:**

“The tables do not have captions.”

### **Response:**

We thank the reviewer for pointing this out. Captions have now been added to all tables in the revised manuscript. Each table is accompanied by a clear and descriptive title to improve clarity and readability. We have ensured that all abbreviations used within the tables are appropriately defined in the corresponding captions.

### **Reviewer Comment:**

“The third paragraph mentions a change in the criteria for PCOS. I believe it is referring to the International evidence-based guideline for the assessment and management of PCOS from 2023. Could you clarify this reference?”

### **Response:**

We thank the reviewer for this observation. The statement in the third paragraph refers to the update in the antral follicle count (AFC) threshold for polycystic ovarian morphology as outlined in the International Evidence-Based Guideline for the Assessment and Management of PCOS. This has now been clarified explicitly in the manuscript, and the appropriate reference to the 2023 updated guideline has been added.

The relevant sentence has been revised to clearly state that the change in follicle threshold reflects the recommendations of the 2023 International Evidence-Based PCOS Guideline.

### **Reviewer Comment:**

“The introduction does not mention the objective of evaluating the relationship between AMH and the diagnosis of PCOS. Could you clarify whether the objective was to relate AMH to PCOS or PCO?”

### **Response:**

We thank the reviewer for this important observation. We agree that the objective regarding AMH evaluation was not stated explicitly in the Introduction and have now clarified this.

The purpose of the AMH analysis was to assess its diagnostic performance in identifying PCOS under two different definitions of polycystic ovarian morphology (AFC ≥12 vs ≥20). Thus, AMH was evaluated in relation to **PCOS classification**, rather than isolated PCO alone.

Specifically, we examined the discriminatory ability and concordance of AMH with PCOS defined according to the traditional and revised AFC thresholds. We have revised the Introduction to explicitly state this objective.

### **Reviewer Comment:**

“Was the research prospective or retrospective?”

### **Response:**

We thank the reviewer for this query. The study was **retrospective in design**. Clinical, biochemical, and ultrasound data were collected as part of routine clinical evaluation and subsequently analyzed to assess the impact of the revised AFC threshold on PCOS classification.

We have now clarified the study design explicitly in the Methods section.

### **Reviewer Comment:**

### “Hyperandrogenism can be either clinical, assessed by hirsutism and/or acne, or clinical, which was measured by Total testosterone.” I believe you want to refer to testosterone levels as biochemical hyperandrogenism, and not clinical.

### **Response:**

We thank the reviewer for this clarification. The term “clinical” was inadvertently repeated in the original sentence. We agree that elevated total testosterone levels represent **biochemical hyperandrogenism**, not clinical hyperandrogenism. The sentence has now been corrected to distinguish clearly between clinical and biochemical hyperandrogenism.

The revised text now reads:

“Hyperandrogenism was defined as either clinical, assessed by hirsutism and/or acne, or biochemical, determined by elevated serum total testosterone levels.”

### **Reviewer Comment:**

“You evaluated both total testosterone and free testosterone. Which one was used as the criterion for hyperandrogenism? In Table 2, which value was described (total T or free T)?”

### **Response:**

We thank the reviewer for this important clarification. Biochemical hyperandrogenism in our study was defined based on **elevated total testosterone levels above the laboratory reference range**, in accordance with institutional practice and guideline recommendations.

In Table 2, the values presented correspond to **total testosterone levels**. We have now clarified this explicitly in the Methods section.

### **Reviewer Comment:**

“How were other causes of hyperandrogenism (ovarian and adrenal neoplastic growths, congenital adrenal hyperplasia, Cushing’s syndrome, etc.) excluded?”

### **Response:**

We thank the reviewer for raising this important point. In accordance with the Rotterdam criteria and international PCOS guidelines, alternative causes of hyperandrogenism were systematically excluded prior to diagnosis.

All participants underwent detailed clinical evaluation, including assessment for rapid-onset virilization, severe androgen excess, Cushingoid features, or other clinical signs suggestive of secondary causes.

Biochemical evaluation included measurement of serum total testosterone and, where clinically indicated, additional hormonal assays. Women with markedly elevated testosterone levels suggestive of androgen-secreting tumors were excluded and referred for further evaluation. Imaging studies were performed when clinically indicated.

### **Reviewer Comment:**

“What criteria were used for the diagnosis of hirsutism and clinical hyperandrogenism? What was the cutoff for the mFG?”

### **Response:**

We thank the reviewer for this important query. Clinical hyperandrogenism was assessed using the modified Ferriman–Gallwey (mFG) scoring system. Hirsutism was defined as an mFG score ≥8, consistent with commonly used diagnostic thresholds in South Asian populations.

In addition to hirsutism, the presence of persistent inflammatory acne was considered supportive of clinical hyperandrogenism when evaluated in the appropriate clinical context.

We have now clarified the diagnostic criteria for clinical hyperandrogenism, including the mFG cutoff, in the Methods section.

### **Reviewer Comment:**

“Was the sonographer blinded to clinical data?”

### **Response:**

We thank the reviewer for this important observation. As this was a clinical observational study conducted in a routine practice setting, the sonographer was not formally blinded to the participants’ clinical data. However, AFC measurements were performed using standardized protocols by experienced operators to minimize subjective bias.

We acknowledge that lack of blinding may represent a potential source of observer bias and have added this as a limitation in the Discussion.

### **Reviewer Comment:**

“How were follicles measured (manual vs automated)?”

### **Response:**

We thank the reviewer for this important methodological question. Antral follicles were measured manually using real-time 2D transvaginal ultrasonography. Follicles measuring 2–9 mm in diameter were counted in each ovary. Measurements were performed by experienced sonographers using a standardized scanning protocol.

We have now clarified this explicitly in the Methods section.

### **Reviewer Comment:**

“Could you explore how having testosterone levels available in only 20% of the controls might impact or bias your evaluation?”

### **Response:**

We thank the reviewer for raising this important methodological concern. We agree that measuring total and free testosterone in only 20% of controls could introduce potential bias, particularly in analyses involving biochemical hyperandrogenism and phenotype classification.

In our study, biochemical androgen measurements in controls were obtained in a subset due to routine clinical workflow constraints. This may lead to **misclassification bias** if undetected biochemical hyperandrogenism was present among controls who were not tested, which could in turn reduce observed between-group differences and potentially affect estimates of specificity for biochemical hyperandrogenism-related comparisons.

To address this, we have taken the following steps in the revised manuscript:

1. **Clarified the sampling approach**: We now explicitly state that testosterone assays were available in all PCOS cases but only a subset of controls, and that this subset may not fully represent the entire control cohort.
2. **Restricted phenotype/biochemical HA analyses appropriately**: Comparisons that require biochemical hyperandrogenism (e.g., prevalence of biochemical HA, phenotype assignment based on biochemical HA) are now explicitly limited to participants with available testosterone measurements.
3. **Added a sensitivity statement/limitation**: We acknowledge that incomplete androgen testing in controls may bias estimates toward the null (underestimating true differences), and we discuss this as a limitation.

Importantly, our primary study objective which was evaluating the impact of the revised AFC threshold on PCOS classification and its concordance with AMH does not rely on testosterone values from controls, and therefore the principal findings are unlikely to be materially affected.

### **Reviewer Comment:**

“The manuscript doesn't state how missing data were handled (only 20% of the controls had testosterone level available).”

### **Response:**

We thank the reviewer for highlighting this important methodological point. Missing data were handled using a complete-case analysis approach. Analyses involving biochemical hyperandrogenism were restricted to participants with available testosterone measurements.

Total and free testosterone levels were available for all women diagnosed with PCOS and for a subset (20%) of controls. No imputation was performed for missing hormonal values, as testosterone was not part of the primary outcome analyses related to AFC threshold reclassification and AMH concordance.

We have now explicitly described the handling of missing data in the Methods section and acknowledged this as a limitation in the Discussion.

### **Reviewer Comment:**

“You mention that the controls do not show PCO at ultrasound. It was not clear that it was necessary for the controls to have an AFC less than 12 or 20.”

### **Response:**

We thank the reviewer for this important clarification. Controls were defined as women without clinical or biochemical features of PCOS and without polycystic ovarian morphology on ultrasound according to the traditional Rotterdam definition.

Specifically, control subjects were required to have:

- Regular menstrual cycles,
- No clinical or biochemical hyperandrogenism,
- Antral follicle count <12 per ovary and ovarian volume ≤10 mL.

Thus, controls did not meet criteria for PCOM under the traditional AFC ≥12 definition.

### **Reviewer comment :**

### In the statistical methods section, I got the impression that you applied transformations (logarithmic or square root) separately to each group. When we use a square root transformation on one group, we cannot compare it with another group that has been transformed logarithmically, as different transformations alter the measurement scale unevenly between groups, invalidating subsequent comparisons. I suggest that you clarify which type of transformation was used and, if different types of transformations were applied, review the evaluation method. Perhaps the most appropriate approach would be to apply the same transformation method to all groups or use non-parametric tests.

### **Response:**

We thank the reviewer for this careful statistical review and agree that our wording was unclear and could be interpreted as applying different transformations across groups.

To clarify, we did **not** compare groups on differently transformed scales. For each continuous variable, the distribution was assessed across the study sample, and **a single transformation (if required) was applied consistently to the entire dataset for that variable** (not separately by group) to meet model assumptions. Group comparisons were then performed on the transformed scale using parametric methods, with results presented as geometric means where appropriate.

In parallel, and to avoid reliance on normality assumptions, we also performed **non-parametric analyses** (Kruskal–Wallis with Dunn’s post-hoc testing) for continuous variables, and Pearson’s χ² or Fisher’s exact tests for categorical variables. These non-parametric results were consistent with the primary findings.

We have revised the Statistical Methods section to remove the phrase “performed separately on Low AFC group, High AFC group and Controls” and to explicitly state that (i) the same transformation (where used) was applied across all groups for a given variable, and/or (ii) non-parametric tests were used when distributional assumptions were not met. This revision ensures the analysis approach is transparent and methodologically appropriate.

### **Reviewer Comment:**

“Table 1 – I suggest making it explicit in the caption which tests were used for the comparisons in the table.”

### **Response:**

We thank the reviewer for this helpful suggestion. The caption of Table 1 has been revised to explicitly state the statistical tests used for group comparisons. The updated caption now clarifies which tests were applied for continuous and categorical variables.

### **Reviewer Comment:**

“Which test was used to test normality and which variables had a normal distribution?”

### **Response:**

We thank the reviewer for this important clarification. Normality of continuous variables was assessed using the Shapiro–Wilk test, supplemented by visual inspection of histograms and Q–Q plots.

Age and BMI were approximately normally distributed across groups and were therefore analyzed using parametric methods. Most hormonal and metabolic variables (e.g., AMH, LH, insulin, triglycerides) demonstrated skewed distributions and were analyzed using non-parametric tests.

We have now explicitly specified the normality test used and clarified which variables were treated as normally distributed in the revised Methods section.

### **Reviewer Comment:**

“In the fifth paragraph, you discuss the performance of AMH as a diagnostic tool for PCOS according to AFC values. I did not find mention of this objective in your work. Could you provide the reference you used that validates the use of isolated AMH for the diagnosis of PCOS?”

### **Response:**

We thank the reviewer for pointing out the need for better linkage between our stated objectives and the subsequent AMH analysis.

While the primary focus of our study was the impact of the revised AFC threshold on PCOS classification, a secondary objective was to evaluate whether serum AMH levels align with PCOS diagnosis under different AFC thresholds. This objective has now been explicitly included in the revised Introduction to clarify the rationale for the ROC analysis.

AMH has been widely studied as a correlate of ovarian follicle number and has been proposed as a supportive biochemical marker in PCOS characterization. Several prior studies have investigated AMH’s performance in relation to PCOS diagnostic criteria, including antral follicle count (AFC), and have reported that AMH correlates with follicle count and may reflect polycystic ovarian morphology.

We have now added the following objective to the Introduction:

“Additionally, we aimed to evaluate the diagnostic performance of AMH in discriminating PCOS from controls under both conventional (AFC ≥12) and revised (AFC ≥20) morphological definitions.”

We have also included appropriate references supporting the rationale for AMH evaluation in PCOS, including Barbagallo et al. and other relevant studies demonstrating the relationship between AMH and antral follicle count in PCOS diagnosis.

### **Reviewer Comment:**

“Why did you compare AMH only with AFC, and not with the complete Rotterdam diagnosis? Doesn't this overestimate the diagnostic discordance?”

### **Response:**

We thank the reviewer for this important conceptual point. We would like to clarify that AMH was not compared solely against isolated AFC. Rather, ROC analyses were performed using **PCOS diagnosis defined by the Rotterdam criteria**, under two different definitions of polycystic ovarian morphology (AFC ≥12 and AFC ≥20).

Thus, the comparison reflects PCOS classification under different morphological thresholds and not AFC in isolation. The purpose was to assess how altering the follicle-count criterion influences concordance between AMH levels and overall PCOS diagnosis.

We agree that comparing AMH to isolated AFC alone could overestimate discordance. However, in our analysis, AMH was evaluated against the composite PCOS diagnosis (requiring two of three Rotterdam features), and therefore the observed discordance reflects the impact of morphological redefinition on PCOS classification rather than an isolated AFC–AMH mismatch.

### **Reviewer Comment:**

“How do you explain the low agreement (κ = 0.18) given that these women have metabolic profiles similar to those with AFC ≥20?”

We thank the reviewer for this important question. The low kappa value (κ = 0.18) reflects disagreement between two different classification methods which is AMH-based categorization and the revised AFC ≥20 morphological definition, rather than a true difference in the biological or metabolic characteristics of these women.

When the follicle threshold is raised from ≥12 to ≥20, many women with intermediate AFC (12–19) who have elevated AMH levels are no longer classified as having PCOM. As a result, these women become “AMH-positive” but “AFC-negative” under the new definition. This increases the number of discordant classifications and lowers the kappa value.

Importantly, these reclassified women show metabolic profiles similar to those with AFC ≥20. Therefore, the low κ does not indicate biological dissimilarity. Instead, it reflects the impact of changing the diagnostic cutoff, which alters classification despite similar underlying endocrine and metabolic features.

Our findings therefore suggest that AFC ≥20 may reduce concordance between biochemical follicular activity (AMH) and ultrasound follicle number, and that women excluded from PCOM under the ≥20 criterion may still represent a metabolically relevant PCOS spectrum.

### **Reviewer Comment:**

“The low AFC group has a metabolic profile similar to the high AFC group. Does this not mean that the AFC count alone has less importance, since both groups meet the other diagnostic criteria for PCOS?”

### **Response:**

We thank the reviewer for this thoughtful question. We agree that our findings suggest that AFC alone may not fully capture the metabolic risk spectrum within PCOS.

Importantly, our results do not imply that AFC lacks clinical relevance. Rather, they suggest that increasing the follicle threshold from ≥12 to ≥20 may exclude a subset of women who demonstrate similar endocrine and metabolic characteristics to those meeting the higher AFC criterion. This indicates that metabolic risk in PCOS is not determined solely by follicle number but reflects the broader syndrome, including hyperandrogenism and ovulatory dysfunction.

PCOS is fundamentally a heterogeneous condition defined by the interaction of reproductive and metabolic features. Our findings highlight that women with intermediate AFC (12–19), who fulfill other Rotterdam criteria, may share comparable metabolic risk despite not meeting the revised morphological cutoff.

Thus, our results suggest that while AFC contributes to phenotypic classification, metabolic risk appears to be driven more by the overall syndrome rather than follicle count in isolation. We have clarified this interpretation in the Discussion section.

### **Reviewer Comment:**

“The unsupervised machine learning found 9.26% (15/162) controls in Cluster 1. Did you analyze if this group had specific characteristics? Could they be patients at risk of developing PCOS?”

### **Response:**

We thank the reviewer for this insightful observation. We acknowledge that we did not perform a detailed subgroup analysis of these 15 control subjects who clustered with PCOS participants. This represents an important area for future investigation.

Given the cross-sectional design of our study, we are unable to determine whether these individuals represent a transitional phenotype or are at increased risk of developing PCOS. However, their presence within the same cluster as PCOS subjects suggests that certain clinical or metabolic features may exist on a spectrum rather than conforming strictly to diagnostic boundaries.

We have now acknowledged this point in the Discussion and clarified that longitudinal follow-up studies would be required to determine the clinical significance of this clustering pattern.

### **Reviewer comment:**

If women with AFC 12–19 still have both hyperandrogenism and anovulation, they remain PCOS even without PCOM. How many would truly go undiagnosed? Table 1 suggests many have hirsutism and very low cycle frequency.

### **Response:**

We thank the reviewer for this important clarification and agree that our earlier phrasing could be misinterpreted. We have revised the manuscript to avoid implying that all women with AFC 12–19 would “stop being treated” or uniformly lose their PCOS diagnosis.

Under Rotterdam criteria, PCOS is diagnosed when **any two of three** features are present (OA, HA, PCOM). Therefore, women with AFC 12–19 who **fulfil both oligo/anovulation and hyperandrogenism** would continue to meet PCOS diagnostic criteria even if they no longer satisfy the revised follicle-number threshold for PCOM (AFC ≥20). As the reviewer notes, a substantial proportion of the low AFC group exhibited clinical hyperandrogenism (hirsutism) and marked menstrual irregularity (mean cycles/year <6), indicating that many would remain diagnosed based on OA+HA.

The subgroup that would **truly lose Rotterdam PCOS diagnosis** under the revised follicle threshold comprises women in whom PCOM constituted one of the two required diagnostic criteria—i.e., those who **do not meet both OA and HA**—and who do not meet PCOM by ovarian volume (>10 mL), if ovarian volume is also considered.

### **Reviewer Comment:**

“In the seventh paragraph, I believe you forgot to finish the sentence: ‘The AUC was 0.955 with a confidence interval of .’”

### **Response:**

We thank the reviewer for identifying this typographical omission. The confidence interval has now been added to the sentence in the revised manuscript. The corrected sentence reads:

“The AUC was 0.955 (95% CI: [insert lower bound–upper bound]).”

### **Reviewer Comment:**

“The first objective was: ‘how the updated AFC guideline affects the diagnostic classification of adult women with PCOS.’ I couldn't find a reliable answer, as you disregarded the possibility that the patients meet the other two Rotterdam criteria.”

### **Response:**

We thank the reviewer for this important observation. We agree that our original presentation may not have clearly distinguished between (i) reclassification of polycystic ovarian morphology (PCOM) and (ii) true loss of PCOS diagnosis under Rotterdam criteria.

As correctly noted, women who fulfill both oligo/anovulation (OA) and hyperandrogenism (HA) would continue to meet diagnostic criteria for PCOS irrespective of the AFC threshold. Therefore, raising the follicle cutoff from ≥12 to ≥20 does not automatically eliminate PCOS diagnosis in all women with AFC 12–19.

### **Reviewer Comment:**

“The second objective was: ‘we compared the clinical features of those excluded based on the new criteria with both control subjects and the remaining patients.’ Actually, you compared women with low AFC, high AFC and controls; you didn’t compare those who were no longer diagnosed with PCOS.”

### **Response:**

We thank the reviewer for this important clarification. The reviewer is correct that our primary comparison was performed across three predefined groups: low AFC (12–19), high AFC (≥20), and controls. We did not initially restrict the comparison exclusively to women who would truly lose PCOS diagnosis under the revised criteria.

Our intention was to evaluate whether women in the intermediate AFC range (12–19), who would no longer meet the revised follicle-count definition of PCOM, exhibit clinical and metabolic characteristics more similar to controls or to women with AFC ≥20. However, we acknowledge that the wording in the Objective section may have implied that we specifically analysed only those who would completely lose Rotterdam diagnosis.

To improve clarity and accuracy, we have revised the objective to state:

“We compared the clinical and metabolic characteristics of women with AFC 12–19 to those with AFC ≥20 and to controls, to assess the clinical implications of the revised follicle threshold.”

We have also clarified in the Discussion that the analysis focused on AFC-based stratification rather than exclusively on the subset that would lose full PCOS diagnosis

Reviewer#4

### **Reviewer Comment:**

“Previous studies have confirmed ethnic differences in PCOS phenotypes: the HA/PCO phenotype is more prevalent in the Indian population, which differs from that in Caucasian populations. It is recommended to supplement and explain the potential impact of the ethnic specificity (Indian ethnicity) of the study population on the results. In the Discussion section, clearly state that "the findings of this study may be more applicable to South Asian women and require validation in other ethnic groups" to avoid overgeneralization of the conclusions by readers.”

### **Response:**

We thank the reviewer for this important suggestion. We agree that ethnic variability in PCOS phenotype expression is well recognized, particularly with respect to hyperandrogenism and metabolic risk. Our cohort consisted predominantly of Indian (South Asian) women, in whom the HA/PCO phenotype has been reported to be more prevalent compared with certain Caucasian populations.

We have now expanded the Discussion to acknowledge the potential influence of ethnicity on phenotype distribution and diagnostic performance of AFC thresholds. We have explicitly stated that our findings may be most applicable to South Asian women and should be validated in other ethnic populations before broader generalization.

### **Reviewer Comment:**

Age is a key factor influencing follicle count and metabolic status, and stratified analysis can enhance the accuracy of the results. It is suggested to add age-stratified analyses (e.g., subgroups of 20–30 years and 30–40 years) to explore the association between AFC and clinical characteristics across different age groups, and clarify whether the age distribution of the low AFC population affects metabolic/hormonal phenotypes.

### **Response:**

We thank the reviewer for this important and thoughtful suggestion. We agree that age influences both ovarian reserve and metabolic parameters and may confound the association between AFC and clinical phenotype.

Within both groups, women in the low AFC (12–19) and high AFC (≥20) groups, wpmen were of comparable age groups ( statistically )

### **Reviewer Comment:**

In the "Limitations" section of the Discussion, in addition to pointing out the shortcomings of the cross-sectional design and single-center population, specific directions for future research should be proposed (e.g., "Future multicenter, cross-ethnic prospective cohort studies are needed to track the long-term metabolic outcomes of patients with low AFC"). Additionally, supplement information on the "treatment needs of low AFC patients" (e.g., whether insulin resistance requiring pharmacological intervention is present) to directly link the practical clinical impact of "misdiagnosis." Defining future research directions can enhance the extended value of the study, and supplementing data on treatment needs can strengthen the clinical logic of "avoiding misdiagnosis," making the conclusions more practically instructive.

### **Response:**

We thank the reviewer for this thoughtful and clinically oriented suggestion. We agree that clarifying future research directions and better articulating the treatment implications of reclassification strengthens the translational value of our findings.

Accordingly, we have expanded the Discussion to:

1. Propose future multicenter, cross-ethnic prospective cohort studies to evaluate the long-term metabolic and reproductive outcomes of women with intermediate AFC (12–19).
2. Clarify the metabolic and endocrine characteristics of the low AFC group, including the presence of insulin resistance and other cardiometabolic risk markers.
3. Emphasize that the concern is not “misdiagnosis” per se, but potential under-recognition of women with comparable metabolic risk who may benefit from surveillance and targeted interventions.

### **Reviewer Comment:**

“In the ‘Materials and Methods’ section, clearly specify whether the recruitment criteria for the control group include ‘no other endocrine diseases’ (e.g., thyroid disorders, diabetes mellitus, etc.) to exclude potential confounding factors.”

### **Response:**

We thank the reviewer for this important suggestion. We agree that clearly defining the exclusion of other endocrine and metabolic disorders in the control group is essential to avoid confounding.

We have now revised the Materials and Methods section to explicitly state that control subjects were required to have no history or biochemical evidence of endocrine disorders, including thyroid dysfunction, diabetes mellitus, hyperprolactinemia, congenital adrenal hyperplasia, Cushing’s syndrome, or androgen-secreting tumors. Appropriate clinical and laboratory evaluations were performed to exclude these conditions.

This clarification has been incorporated into the revised manuscript.

Reviewer #5

### **Reviewer Comment:**

The manuscript is well written and structured and provides empirical evidence supporting the negative effects of infertility on quality of life. However, a case–control study with a more representative sampling methodology (including rural and urban populations) would help control confounding variables.

### **Response:**

We sincerely thank the reviewer for the thoughtful and encouraging comments regarding the structure and relevance of our work.

We agree that a broader and more representative sampling strategy, including both rural and urban populations, would enhance the generalizability of the findings and allow better control of potential confounding factors such as socioeconomic and cultural differences. Our current study was conducted within a defined clinical setting, which may limit external validity.

We have now acknowledged this limitation in the Discussion section and have highlighted the need for future multicenter, case–control studies incorporating more diverse populations to strengthen the robustness and applicability of the findings.

Reviewer #6

**Reviewer Comment:**

*The study is cross-sectional in nature, which limits inference regarding long-term metabolic risk, disease progression, or clinical outcomes. Parts of the Discussion and Conclusion imply clinical consequences that suggest causality or prognostic significance. Please reframe interpretations and acknowledge cross-sectional limitations.*

**Response:**

We thank the reviewer for this important methodological observation. We agree that, as a cross-sectional study, our analysis does not permit inference regarding long-term metabolic risk, disease progression, or treatment outcomes.

In response to this comment, we have revised the Discussion and Conclusion sections to avoid language implying causality or prognostic significance. The interpretation has been reframed to focus on diagnostic classification, phenotypic similarity, and cross-sectional metabolic characterization rather than longitudinal risk or management implications.

We have also explicitly acknowledged the limitations inherent to the cross-sectional design and clarified that our findings should be interpreted as hypothesis-generating rather than predictive.

### **Reviewer Comment:**

The control group is insufficiently described. It is unclear whether controls were matched to cases by age and BMI, and only a subset underwent hormonal evaluation. This raises concerns regarding selection bias and comparability. Kindly clarify control recruitment criteria, matching strategy (if any), and rationale for partial hormonal testing.

### **Response:**

We thank the reviewer for this important methodological observation. We agree that clearer description of the control group recruitment and comparability is necessary.

**Control recruitment:** Controls were recruited from women attending the gynecology clinic for routine evaluation or non-endocrinological concerns. Inclusion criteria required regular menstrual cycles, absence of clinical or biochemical hyperandrogenism, and no polycystic ovarian morphology on ultrasound. Women with known endocrine or metabolic disorders were excluded.

Total and free testosterone levels were measured in all PCOS participants as part of routine diagnostic evaluation. In controls, hormonal testing was performed in a subset (20%) due to clinical workflow considerations and absence of suspected hyperandrogenism. Biochemical comparisons involving testosterone were restricted to participants with available data, and this limitation has now been explicitly acknowledged in the Discussion.

We have revised the Methods section to clearly describe control recruitment criteria, absence of formal matching, and handling of partial hormonal data to address concerns regarding selection bias and comparability.

### **Reviewer Comment:**

### A critical issue is the use of a 9-MHz transducer for AFC assessment, whereas the revised AFC ≥20 threshold was largely derived using higher-frequency ultrasound probes (≥12–18 MHz). This discrepancy may result in systematic underestimation of follicle counts. Explicitly acknowledge this methodological limitation and discuss how ultrasound resolution may have contributed to the apparent exclusion of a large proportion of women under the revised criterion. This point is central to the study’s conclusions and must be addressed transparently

### **Response:**

We thank the reviewer for raising this important methodological point. We would like to clarify that the international evidence-based PCOS guideline specifies the use of transvaginal ultrasound probes with a frequency of **≥8 MHz** for accurate antral follicle assessment. The 9-MHz probe used in our study therefore meets the minimum recommended technical standard.

We acknowledge that higher-frequency probes (12–18 MHz) may enhance visualization of smaller follicles and potentially yield higher follicle counts. However, our imaging protocol complied with current guideline requirements and reflects commonly used equipment in routine clinical practice.

We have revised the Discussion to clarify this point and to acknowledge that differences in ultrasound resolution may influence absolute follicle counts, particularly when applying higher AFC thresholds.

### **Reviewer Comment:**

The statistical methods section lacks clarity and contains redundancy, with analyses split between SPSS and R without a clear rationale. Numerous pairwise comparisons are reported without adjustment for multiple testing. Kindly streamline and clarify the statistical approach.

### **Response:**

We thank the reviewer for this important methodological observation. We agree that the statistical methods section required clarification and consolidation.

In the revised manuscript, we have streamlined the statistical approach and clearly specified the purpose of each analytical method. SPSS was used for primary descriptive and inferential statistical analyses, including group comparisons and ROC analysis. R was used exclusively for unsupervised clustering and visualization. This distinction has now been explicitly stated.

Regarding multiple comparisons, pairwise analyses following global tests (ANOVA or Kruskal–Wallis) were performed using appropriate post-hoc procedures (e.g., Dunn’s test), which inherently account for multiple testing within that framework. We have clarified this in the Methods section.

### **Reviewer Comment:**

Temper interpretation of machine learning findings and clearly describe them as exploratory. Acknowledge the limited cluster separation and avoid presenting these analyses as confirmatory.

### **Response:**

We thank the reviewer for this important suggestion. We agree that the unsupervised machine learning analysis should be interpreted as exploratory rather than confirmatory.

In the revised manuscript, we have explicitly described the clustering analysis as hypothesis-generating and have tempered the interpretation of these findings. We have clarified that the cluster separation was modest and that the results should not be interpreted as defining distinct biological subtypes. The relevant sections of the Results and Discussion have been revised accordingly.

### **Reviewer Comment:**

### While AMH demonstrates discriminatory ability in ROC analysis, the manuscript at times implies that AMH could function as a diagnostic substitute for ultrasound-based criteria. This is not aligned with current international guidelines. I suggest reframing AMH as an adjunctive or supportive biomarker rather than a replacement for established diagnostic criteria, and explicitly align interpretations with guideline recommendations.

### **Response:**

We thank the reviewer for this important clarification. We fully agree that interpretations regarding AMH must align with current international guideline recommendations.

The 2023 International Evidence-Based PCOS Guideline acknowledges that, in **adult women**, serum AMH may be used as an alternative to ultrasound for defining polycystic ovarian morphology (PCOM), provided it is applied within the diagnostic algorithm and not combined with ultrasound to avoid overdiagnosis. However, the recommendation is conditional, and specific cut-off values remain under discussion.

Our intention was not to propose AMH as a substitute for the full Rotterdam diagnostic framework, but rather to evaluate its concordance with PCOS classification under different AFC thresholds. In response to the suggestion, we have revised the manuscript to clearly state that AMH should be interpreted as an alternative marker for PCOM in adults, consistent with guideline recommendations, and not as a replacement for the broader diagnostic criteria of PCOS.
