# Supplementary material for: Impact of revised antral follicle count threshold on PCOS diagnosis: A comparative clinical study
Source: PLoS One. 2026 Oct 15;21(10):e0358554. doi: 10.1371/journal.pone.0358554 (PMC13671520; doi:10.1371/journal.pone.0358554)
Supplement: Supplementary file 3 [file pone.0358554.s005.docx]

**Reviewer comment:**
Please spell out the abbreviation AMH when first mentioned.

Still, the authors claim: “Practically, the data indicate that using ≥20 follicles as a diagnostic cut-point would reclassify 62% of symptomatic women as ‘non-PCOS’ despite AMH levels consistent with ovarian hyperactivity.”

Please make a table that indicates:
a) Phenotypes with the cut-off of 12
b) Phenotypes with the cut-off of 20 and how many women would lose PCOS diagnosis

Then you can answer the question of how many women would be left undiagnosed if the cut-off of 12 is utilized.

**Response:**
We thank the reviewer for this important comment. We have now spelled out **anti-Müllerian hormone (AMH)** at its first mention in the manuscript.

We agree that our previous statement that increasing the follicle-count threshold to ≥20 would reclassify approximately 62% of symptomatic women as “non-PCOS” was imprecise. Loss of the polycystic ovarian morphology criterion does not necessarily imply loss of the PCOS diagnosis, because women fulfilling both the oligo-anovulation and hyperandrogenism criteria continue to meet the Rotterdam diagnostic criteria.

We therefore performed a phenotype-wise reclassification using an antral follicle count threshold of ≥12 and subsequently ≥20 follicles. The revised phenotype distribution and reclassification are presented in the following table.

| **Original phenotype using AFC ≥12** | **n (%)** | **Classification after applying AFC ≥20** | **n (%)** |
| --- | --- | --- | --- |
| A: OA + HA + PCOM | 190 (56.7) | A: OA + HA + PCOM | 62 (18.5) |
|  |  | B: OA + HA | 128 (38.2) |
| B: OA + HA | 13 (3.9) | B: OA + HA | 13 (3.9) |
| C: HA + PCOM | 104 (31.0) | C: HA + PCOM | 32 (9.6) |
|  |  | Hyperandrogenism only | 72 (21.5) |
| D: OA + PCOM | 28 (8.4) | Oligo-anovulation only | 28 (8.4) |
| **No longer fulfilling the Rotterdam criteria** | — |  | **100 (29.9)** |

Abbreviations: AFC, antral follicle count; HA, hyperandrogenism; OA, oligo-anovulation; PCOM, polycystic ovarian morphology; PCOS, polycystic ovary syndrome.

Among the 190 women with phenotype A at the AFC ≥12 threshold, 128 had an AFC of 12–19 and therefore lost the PCOM criterion when the threshold was increased to ≥20. However, these women continued to fulfil both the oligo-anovulation and hyperandrogenism criteria and were reclassified as phenotype B. They therefore retained the diagnosis of PCOS.

Among the 104 women with phenotype C, 72 lost the PCOM criterion and were reclassified as having isolated hyperandrogenism. Similarly, all 28 women with phenotype D lost the PCOM criterion and were reclassified as having isolated oligo-anovulation. These women no longer fulfilled at least two of the three Rotterdam criteria.

Consequently, **100 of the 335 women originally diagnosed with PCOS using AFC ≥12, corresponding to 29.9%, would no longer fulfil the Rotterdam criteria when the AFC threshold was increased to ≥20**. The remaining **235 women (70.1%)** would retain the diagnosis of PCOS.

We have therefore replaced the previous statement with the following in the Results section:

“Increasing the AFC threshold for polycystic ovarian morphology from ≥12 to ≥20 follicles resulted in loss of the PCOM criterion in 228 of 335 women (68.1%). Of these, 128 women originally classified as phenotype A continued to fulfil the oligo-anovulation and hyperandrogenism criteria and were reclassified as phenotype B, while 62 remained phenotype A. All 13 women with phenotype B remained unchanged. Among women with phenotype C, 72 lost the PCOM criterion and were reclassified as having isolated hyperandrogenism, while 32 remained phenotype C. All 28 women with phenotype D were reclassified as having isolated oligo-anovulation. Overall, 100 of 335 women (29.9%) no longer fulfilled the Rotterdam diagnostic criteria for PCOS after applying the AFC ≥20 threshold.”

The changes made in the discussion as well

“From a practical standpoint, increasing the diagnostic threshold for polycystic ovarian morphology from ≥12 to ≥20 follicles would result in 29.9% of women no longer meeting the Rotterdam criteria for PCOS. Given that many of these women continued to demonstrate biochemical evidence of ovarian hyperactivity, including elevated AMH levels, these findings raise concerns that the revised threshold may reduce diagnostic sensitivity in symptomatic women with less marked follicular excess.”

**Reviewer #5:**
The manuscript has undergone a significant improvement after revision, particularly in acknowledging its limitations and clarifying the interpretation of AMH. The discussion appropriately refrains from asserting that AMH serves as a replacement diagnostic criterion.

The manuscript alternates between “cross-sectional” and “retrospective observational”, which are unfortunately not interchangeable. The study should be best described as a retrospective cross-sectional observational study. I suggest that the researchers ensure consistency in their terminology.

**Response:**
We thank the reviewer for the positive comments on our revisions and for this helpful suggestion. We agree that the terminology should be consistent throughout the manuscript. Accordingly, we have revised the manuscript to describe the study uniformly as a **retrospective cross-sectional observational study** in the Abstract, Methods, and elsewhere in the manuscript, replacing instances where only "cross-sectional" or "retrospective observational" had been used. This terminology more accurately reflects the study design and has been applied consistently throughout the revised manuscript.
