# Supplementary material for: Impact of revised antral follicle count threshold on PCOS diagnosis: A comparative clinical study
Source: PLoS One. 2026 Oct 15;21(10):e0358554. doi: 10.1371/journal.pone.0358554 (PMC13671520; doi:10.1371/journal.pone.0358554)
Supplement: Peer review history

# Peer review: Decision Letter 0

**DOI:** 10.1371/journal.pone.0358554.r001

**Authors:** Ahmed Maged

29 Dec 2025

Dear Dr. Malhotra,

- **Please respond to all reviewers comments.**

plosone@plos.org. When you're ready to submit your revision, log on to https://www.editorialmanager.com/pone/ and select the 'Submissions Needing Revision' folder to locate your manuscript file.

- A letter that responds to each point raised by the academic editor and reviewer(s). You should upload this letter as a separate file labeled 'Response to Reviewers'.
- A marked-up copy of your manuscript that highlights changes made to the original version. You should upload this as a separate file labeled 'Revised Manuscript with Track Changes'.
- An unmarked version of your revised paper without tracked changes. You should upload this as a separate file labeled 'Manuscript'.

If you would like to make changes to your financial disclosure, please include your updated statement in your cover letter. Guidelines for resubmitting your figure files are available below the reviewer comments at the end of this letter.

If applicable, we recommend that you deposit your laboratory protocols in protocols.io to enhance the reproducibility of your results. Protocols.io assigns your protocol its own identifier (DOI) so that it can be cited independently in the future. For instructions see: https://journals.plos.org/plosone/s/submission-guidelines#loc-laboratory-protocols. Additionally, PLOS ONE offers an option for publishing peer-reviewed Lab Protocol articles, which describe protocols hosted on protocols.io. Read more information on sharing protocols at https://plos.org/protocols?utm_medium=editorial-email&utm_source=authorletters&utm_campaign=protocols.

We look forward to receiving your revised manuscript.

Kind regards,

Ahmed Mohamed Maged, MD

Academic Editor

PLOS One

**Journal Requirements:**

1. When submitting your revision, we need you to address these additional requirements.

Please ensure that your manuscript meets PLOS ONE's style requirements, including those for file naming. The PLOS ONE style templates can be found at

https://journals.plos.org/plosone/s/file?id=wjVg/PLOSOne_formatting_sample_main_body.pdf and

https://journals.plos.org/plosone/s/file?id=ba62/PLOSOne_formatting_sample_title_authors_affiliations.pdf

2. Please include your tables as part of your main manuscript and remove the individual files. Please note that supplementary tables (should remain/ be uploaded) as separate "supporting information" files.

3. In the online submission form, you indicated that “Data shall be available from the corresponding author on a reasonable request.”

All PLOS journals now require all data underlying the findings described in their manuscript to be freely available to other researchers, either a. In a public repository, b. Within the manuscript itself, or c. Uploaded as supplementary information.

This policy applies to all data except where public deposition would breach compliance with the protocol approved by your research ethics board. If your data cannot be made publicly available for ethical or legal reasons (e.g., public availability would compromise patient privacy), please explain your reasons on resubmission and your exemption request will be escalated for approval.

4. If the reviewer comments include a recommendation to cite specific previously published works, please review and evaluate these publications to determine whether they are relevant and should be cited. There is no requirement to cite these works unless the editor has indicated otherwise.

[Note: HTML markup is below. Please do not edit.]

Reviewers' comments:

**Comments to the Author**

1. Is the manuscript technically sound, and do the data support the conclusions?

Reviewer #1: Yes

Reviewer #2: Yes

Reviewer #3: Partly

Reviewer #4: Yes

Reviewer #5: Yes

Reviewer #6: Partly

**********

2. Has the statistical analysis been performed appropriately and rigorously?

Reviewer #1: Yes

Reviewer #2: Yes

Reviewer #3: No

Reviewer #4: Yes

Reviewer #5: Yes

Reviewer #6: Yes

**********

3. Have the authors made all data underlying the findings in their manuscript fully available?

The PLOS Data policy

Reviewer #1: Yes

Reviewer #2: Yes

Reviewer #3: No

Reviewer #4: Yes

Reviewer #5: Yes

Reviewer #6: Yes

**********

4. Is the manuscript presented in an intelligible fashion and written in standard English?

Reviewer #1: Yes

Reviewer #2: Yes

Reviewer #3: Yes

Reviewer #4: Yes

Reviewer #5: Yes

Reviewer #6: Yes

**********

**Reviewer #1:**  The study focuses on the debate on the optimal AFC cut-off for PCOM diagnosis. The question is rather important and also touches the issue of optimal AMH cutoff for PCOM diagnosis. Nevertheless, the background should be better described in the Introduction. Where do the AFC cutoffs of 12 (authors call this the “old” cutoff) and 20 originate and why is it important to study this? The authors seem to assume the reader is familiar with the issue. Also, they later talk about NIH criteria, without describing what this means. Please improve the Introduction.

For example: “PCO is defined as either 12 or more antral follicles measuring 2–9 mm in diameter or an increased ovarian volume > 10 cm2 in either ovary.”

Please indicate the source of the definition. The current guideline does recommend 20 or more. The authors should discuss the issue of 12 vs. 20 in a sentence, as a few chapter later they say that “the revised AFC threshold of 20 could lead to a significant number of patients being missed”. Where is the number 12 originated (REF) and where is the revision of 20 introduced (REF)? And some background on why the change was made (improvement of ultrasound).

Where is the statement “Since PCO is the most commonly used parameter for diagnosing PCOS” derived from? In the updated guideline, the first step is to assess irregular cycles and hyperandrogenism, and the diagnosis can be made based on these, without assessment of PCOM. PCOM assessment is introduced only as a third step.

The choice of serum measurement panel is valid. Testosterone measurement by RIA is not optimal, as the sensitivity is not often good enough for women, LC-MS should be preferred. Also, the assays should be better described; for example, now it is not clear how the assay estimate free T (vs. total T).

Specific questions:

1. Does the Beckman Coulter AMH kit mean the Gen II? Please describe better, so that the results can be compared to previous studies. Also, remember to mention the assay used when comparing to previous studies. See Barbagallo et al (JCEM 2020, https://doi.org/10.1210/clinem/dgae153).

2. How many of the low AFC group fulfill the volume > 10 mm2 criterion? Could the authors provide also the mean volumes?

3. The authors say 336 women would have missed the diagnosis if the AFC cutoff of 20 had been used. So, these 336 women would only have irregular cycles or hyperandrogenism? How about ovarian volume in these 336 women? What is the mean age of the women who would miss PCOS diagnosis?

4. How do the authors explain that the cutoff of AMH is higher for the lower AFC group than for the higher one?

5. Did the authors consider age when assessing AMH and AFC? For example, Barbagallo et al (JCEM 2020, https://doi.org/10.1210/clinem/dgae153) have noted, that in younger PCOS women (<30) optimal cutoff for AFC 12 is 5,5 ng/mL, and for PCOS cases >30 years, 5,5 ng/mL.

6. Do the authors think the AFC cutoff should be ethnicity-dependent, like FG-scores are for clinical hyperandrogenism?

**Reviewer #2:**  Improvements such as adding citation numbers in the text as well as a few improvements in English.

TITLE: The title should more closely reflect the contents of the study: Impact of Revised Antral Follicle Count Threshold on PCOS Diagnosis: A Comparative Clinical Study ---->

Revised Title:

Increase in Follicle Threshold required for PCOS diagnosis excluded 62% of women diagnosed with PCOS using Rotterdam Criteria in India

ABSTRACT: When talking about people, i think rounding to the closest whole number is preferable to saying 62.2%: --> improved version might state:

208 (62%) of the 335 women had AFC between 12 and 19 and would be excluded under new guidelines.

"ovarian volume > 10 cm2." Most likely the authors meant: > 10 mL2 ??

"this AMENDMENT needs to be highlighted"---> Did the authors mean 'this ITEM needs to be '?

July, 2018 and June, 2019.---> Why did it take 6 1/2 years to submit ?

"Hyperandrogenism can be either clinical, assessed by hirsutism and/or acne, or clinical, which was measured by

Total testosterone." --> Hyperandrogenism can be either clinical, assessed by hirsutism and/or acne, or defined by Total testosterone level in lab results.

" (75-g oral glucose tolerance test) were WITHDRAWN" ---> were drawn

Results "Coming to the comparison between the controls and low AFC group, who have been

exempted from PCOS as per the latest recommendations, we observed some noteworthy

differences." ---> Comparing the controls to the low AFC group who would have been exempted from PCOS as per......

DISCUSSION:

"Rotterdam criteria considered women with AFC > 12 to be PCO, and therefore they needed either OA or HA to be diagnosed as PCOS and thus managed accordingly." -----> Rotterdam criteria define PCOS as women with AFC > 12 accompanied by either OA or HA.

"On the contrary, patients with AFC between 12-19, who were earlier managed as PCOS, will be treated as normal as per the international guideline development group." ------> According to the new AFC cut off of 19, patients with AFC between 12-19, who were earlier managed as PCO, will be treated as normal.....

"Jonard in 2003" ---> Add number of the Jonard Citation (11)

"Another study conducted by Chen et al.," Add number of the citation of Chen (12)

"A study was conducted across 5 different zones of India to find prevalence of PCOS and

the phenotypic spectrum of PCOS." Add citation number (2)

"In another Indian prospective study byKar et al on various types of phenotypes suggested

a cut-off of 12 and 10 follicle numbers per ovary ---> add citation (5) and add AFC: In another Indian prospective study by Kar et. al. (5) on various types of phenotypes suggested a cut-off of 12 AFC and 10 follicles per ovary in

"A pilot study done in the North African continent aimed to validate follicular and ovarian

thresholds by an 18-MHz ultrasound imaging in polycystic ovary syndrome.-------> Needs citation= (15)--->

A pilot study done in the North African continent aimed to validate follicular and ovarian

thresholds by an 18-MHz ultrasound imaging in polycystic ovary syndrome found......(15 ).

**Reviewer #3:**  1. General issues

- Data availability does not fully meet PLOS’s “without restriction” standard. I suggest making the data available in a public repository.

- The references are not marked in the text.

- The tables do not have captions.

- I separated my questions and suggestions by sections of the article.

-

2. Introduction

Question: The third paragraph mentions a change in the criteria for PCOS. I believe it is referring to the International evidence-based guideline for the assessment and management of polycystic ovary syndrome (PCOS) from 2023. Could you clarify this reference?

Suggestion: The introduction does not mention the objective of evaluating the relationship between AMH and the diagnosis of PCOS. I suggest adding this objective, since this evaluation was conducted. Latter, I have comments about this evaluation. Could you clarify whether the objective was to relate AMH to PCOS or PCO?

3. Methods

Question: Was the research prospective or retrospective?

Question: “Hyperandrogenism can be either clinical, assessed by hirsutism and/or acne, or clinical, which was measured by Total testosterone.” I believe you want to refer to testosterone levels as biochemical hyperandrogenism, and not clinical.

Question: You evaluated both total testosterone and free testosterone, which one was used as the criterion for hyperandrogenism? In Table 2, which value was described (total T or free T)?

Question: How were other causes of hyperandrogenism (ovarian and adrenal neoplastic growths, congenital adrenal hyperplasia, Cushing’s syndrome, etc.) excluded?

Question: What criteria were used for the diagnosis of hirsutism and clinical hyperandrogenism? What was the cutoff for the mFG?

Question: Was the sonographer blinded to clinical data?

Question: How were follicles measured (manual vs automated)?

Question: Could you explore how having testosterone levels available in only 20% of the controls might impact or bias your evaluation?

Question: The manuscript doesn't state how missing data were handled (only 20% of the controls had testosterone level available).

Question: You mention that the controls do not show PCO at ultrasound. It was not clear that it was necessary for the controls to have an AFC less than 12 or 20.

4. Statistical Analysis

Question: In the statistical methods section, I got the impression that you applied transformations (logarithmic or square root) separately to each group. When we use a square root transformation on one group, we cannot compare it with another group that has been transformed logarithmically, as different transformations alter the measurement scale unevenly between groups, invalidating subsequent comparisons. I suggest that you clarify which type of transformation was used and, if different types of transformations were applied, review the evaluation method. Perhaps the most appropriate approach would be to apply the same transformation method to all groups or use non-parametric tests.

5. Results

Question: Table 1 - I suggest making it explicit in the caption which tests were used for the comparisons in the table.

Question: Which test was used to test normality and which variables had a normal distribution?

Question: In the fifth paragraph, you discuss the performance of AMH as a diagnostic tool for PCOS according to AFC values. I did not find mention of this objective in your work. Could you provide the reference you used that validates the use of isolated AMH for the diagnosis of PCOS?

Question: Why did you compare AMH only with AFC, and not with the complete Rotterdam diagnosis? Doesn't this overestimate the diagnostic discordance?

Question: How to explain the low agreement (κ=0.18) with the fact that these women have metabolic profiles similar to those with AFC≥20?

Question: The low AFC group has a metabolic profile similar to the high AFC group, does this not mean that the AFC count alone has less importance, since both groups meet the other diagnostic criteria for PCOS?

Question: The unsupervised machine learning found 9.26% (15/162) controls in Cluster 1. Did you analyze if this group had specific characteristics? Could they be patients at risk of developing PCOS?

6. Discusion

Question: You state that all patients with PCOS according to the Rotterdam criteria with AFC between 12 and 20 would stop being treated; however, if this group meets the other two criteria (hyperandrogenism and anovulation), they will remain with the PCOS diagnosis. How many subjects in the study would really go undiagnosed with PCOS? In Table 1, it seems to me that almost half of the low AFC group presents hirsutism and the average number of cycles per year was less than 6.

Question: In the seventh paragraph, I believe you forgot to finish the sentence: “The AUC was 0.955 with a confidence interval of .”.

Question: The first objective was: "how the updated AFC guideline affects the diagnostic classification of adult women with PCOS." I couldn't find a reliable answer, as you disregarded the possibility that the patients meet the other two Rotterdam criteria.

Question: The second objective was: “we compared the clinical features of those excluded based on the new criteria with both control subjects and the remaining patients.”. Actually, you compared women with low AFC, high AFC and controls, you didn't compare those who were no longer diagnosed with PCOS.

7. Comments

I believe the topic is very relevant and I found the idea of conducting a profile assessment of patients who no longer have the PCOS diagnosis due to the change in the AFC cutoff interesting.

However, I believe that the methodology used did not achieve what was proposed. Women in the Low AFC group exhibit a high rate of hirsutism and most likely the majority have anovulatory cycles (Table 1), therefore, not all would be undiagnosed with PCOS.

I believe that with the suggested adjustments, the publication will add very important information for the scientific community and can impact the care of women with PCOS.

**Reviewer #4:**  Thank you for submitting your research paper entitled Impact of Revised Antral Follicle Count Threshold on PCOS Diagnosis: A Comparative Clinical Study. This study focuses on the clinical implications of the revised antral follicle count (AFC) threshold in the diagnosis of polycystic ovary syndrome (PCOS), with a research topic that holds both practical significance and academic value. The study design is generally rigorous, and the data support is relatively sufficient. Based on a comprehensive analysis of the full text, the following constructive revision suggestions are proposed to assist you in improving the paper:

Previous studies have confirmed ethnic differences in PCOS phenotypes: the HA/PCO phenotype is more prevalent in the Indian population, which differs from that in Caucasian populations. It is recommended to supplement and explain the potential impact of the ethnic specificity (Indian ethnicity) of the study population on the results. In the Discussion section, clearly state that "the findings of this study may be more applicable to South Asian women and require validation in other ethnic groups" to avoid overgeneralization of the conclusions by readers.

Age is a key factor influencing follicle count and metabolic status, and stratified analysis can enhance the accuracy of the results. It is suggested to add age-stratified analyses (e.g., subgroups of 20–30 years and 30–40 years) to explore the association between AFC and clinical characteristics across different age groups, and clarify whether the age distribution of the low AFC population affects metabolic/hormonal phenotypes.

In the "Limitations" section of the Discussion, in addition to pointing out the shortcomings of the cross-sectional design and single-center population, specific directions for future research should be proposed (e.g., "Future multicenter, cross-ethnic prospective cohort studies are needed to track the long-term metabolic outcomes of patients with low AFC"). Additionally, supplement information on the "treatment needs of low AFC patients" (e.g., whether insulin resistance requiring pharmacological intervention is present) to directly link the practical clinical impact of "misdiagnosis." Defining future research directions can enhance the extended value of the study, and supplementing data on treatment needs can strengthen the clinical logic of "avoiding misdiagnosis," making the conclusions more practically instructive.

In the "Materials and Methods" section, clearly specify whether the recruitment criteria for the control group include "no other endocrine diseases" (e.g., thyroid disorders, diabetes mellitus, etc.) to exclude potential confounding factors.

This study addresses a topic of significant clinical value, with reliable core findings that provide critical evidence for the optimization of PCOS diagnostic criteria. It is recommended that the authors focus on revising and improving the paper in four aspects: "population representativeness, methodological details, statistical rigor, and result visualization" to further enhance the scientific rigor, reproducibility, and clinical guiding significance of the paper. After revisions, the paper is expected to meet the publication requirements of PLOS ONE and provide valuable references for clinical practice and guideline development in related fields.

**Reviewer #5:**  Thank you for the invitation to review this article. This research article is not only well written and structured, but it seems to provide some empirical evidence to support the generally accepted view on the negative effects of infertility on the quality of life of the affected couple. Although the authors adopted the use of a validated questionnaire adapted to the local culture, a case-control study with a more representative sampling methodology (rural and urban populations) would help in controlling some of the confounding variables identified in the study.

**Reviewer #6:**  1. The study is cross-sectional in nature, which limits inference regarding long-term metabolic risk, disease progression, or clinical outcomes. Despite this, parts of the Discussion and Conclusion imply clinical consequences that suggest causality or prognostic significance. I suggest reframing interpretations to emphasize diagnostic classification and phenotypic similarity rather than longitudinal risk or management implications. Also, Kindly acknowledge the limitations of cross-sectional data.

2.The control group is insufficiently described. It is unclear whether controls were matched to cases by age and BMI, and only a subset underwent hormonal evaluation. This raises concerns regarding selection bias and comparability. Kindly

Clarify control recruitment criteria, matching strategy (if any), and rationale for partial hormonal testing.

3. A critical issue is the use of a 9-MHz transducer for AFC assessment, whereas the revised AFC ≥20 threshold was largely derived using higher-frequency ultrasound probes (≥12–18 MHz). This discrepancy may result in systematic underestimation of follicle counts. Explicitly acknowledge this methodological limitation and discuss how ultrasound resolution may have contributed to the apparent exclusion of a large proportion of women under the revised criterion. This point is central to the study’s conclusions and must be addressed transparently.

4. The statistical methods section lacks clarity and contains redundancy, with analyses split between SPSS and R without a clear rationale. Numerous pairwise comparisons are reported without adjustment for multiple testing. Kindly

Streamline and clarify the statistical approach, specifying the purpose of each analytical method.

5. Temper interpretation of machine learning findings and clearly describe them as exploratory. Acknowledge the limited cluster separation and avoid presenting these analyses as confirmatory.

6. While AMH demonstrates discriminatory ability in ROC analysis, the manuscript at times implies that AMH could function as a diagnostic substitute for ultrasound-based criteria. This is not aligned with current international guidelines. I suggest

reframing AMH as an adjunctive or supportive biomarker rather than a replacement for established diagnostic criteria, and explicitly align interpretations with guideline recommendations.

**********

what does this mean?). If published, this will include your full peer review and any attached files.

If you choose “no”, your identity will remain anonymous but your review may still be made public.

**Do you want your identity to be public for this peer review?**  For information about this choice, including consent withdrawal, please see our Privacy Policy

Reviewer #1: No

Reviewer #2: **Yes:**  Judy Slome Cohain

Reviewer #3: No

Reviewer #4: No

Reviewer #5: **Yes:**  Ekwuazi Kingsley Emeka

Reviewer #6: **Yes:**  Michael Sylvester Archibong

**********

[NOTE: If reviewer comments were submitted as an attachment file, they will be attached to this email and accessible via the submission site. Please log into your account, locate the manuscript record, and check for the action link "View Attachments". If this link does not appear, there are no attachment files.]

To ensure your figures meet our technical requirements, please review our figure guidelines: https://journals.plos.org/plosone/s/figures

You may also use PLOS’s free figure tool, NAAS, to help you prepare publication quality figures: https://journals.plos.org/plosone/s/figures#loc-tools-for-figure-preparation.

NAAS will assess whether your figures meet our technical requirements by comparing each figure against our figure specifications.

# Author comment: Author response to Decision Letter 1

**DOI:** 10.1371/journal.pone.0358554.r002

6 Apr 2026

Reviewer Comment:

“Where do the AFC cutoffs of 12 (‘old’) and 20 originate and why is it important to study this? The authors assume familiarity. NIH criteria are also mentioned without explanation. Please improve the Introduction.”

Response

We thank the reviewer for this insightful comment. We agree that the background regarding the evolution of AFC thresholds for polycystic ovarian morphology (PCOM) and the diagnostic criteria for PCOS was insufficiently detailed in the original submission.

To address this:

1. We have now clearly described:

o The origin of the AFC ≥12 threshold from the 2003 Rotterdam consensus.

o The rationale for revising this threshold to AFC ≥20 in the 2023 International PCOS Guideline, primarily due to advancements in ultrasound resolution.

2. We have elaborated on:

o The diagnostic frameworks of NIH (1990) and Rotterdam (2003) criteria.

o How the inclusion of PCOM as a diagnostic criterion created phenotype heterogeneity.

3. We have clarified:

o Why reassessing AFC thresholds is clinically relevant, especially in reproductive medicine populations where ovarian reserve markers directly influence stimulation strategies, prognosis, and risk stratification (e.g., OHSS).

These changes have been incorporated into the revised Introduction.

Attachment Submitted filename: Response to reviewers (1).docx

# Peer review: Decision Letter 1

**DOI:** 10.1371/journal.pone.0358554.r003

**Authors:** Ricardo Cobucci

22 Apr 2026

Dear Dr. Malhotra,

Please submit your revised manuscript by Jun 06 2026 11:59PM. If you will need more time than this to complete your revisions, please reply to this message or contact the journal office at plosone@plos.org. When you're ready to submit your revision, log on to https://www.editorialmanager.com/pone/ and select the 'Submissions Needing Revision' folder to locate your manuscript file.

- A letter that responds to each point raised by the academic editor and reviewer(s). You should upload this letter as a separate file labeled 'Response to Reviewers'.
- A marked-up copy of your manuscript that highlights changes made to the original version. You should upload this as a separate file labeled 'Revised Manuscript with Track Changes'.
- An unmarked version of your revised paper without tracked changes. You should upload this as a separate file labeled 'Manuscript'.

If you would like to make changes to your financial disclosure, please include your updated statement in your cover letter. Guidelines for resubmitting your figure files are available below the reviewer comments at the end of this letter.

If applicable, we recommend that you deposit your laboratory protocols in protocols.io to enhance the reproducibility of your results. Protocols.io assigns your protocol its own identifier (DOI) so that it can be cited independently in the future. For instructions see: https://journals.plos.org/plosone/s/submission-guidelines#loc-laboratory-protocols. Additionally, PLOS ONE offers an option for publishing peer-reviewed Lab Protocol articles, which describe protocols hosted on protocols.io. Read more information on sharing protocols at https://plos.org/protocols?utm_medium=editorial-email&utm_source=authorletters&utm_campaign=protocols.

As the corresponding author, your ORCID iD is verified in the submission system and will appear in the published article. PLOS supports the use of ORCID, and we encourage all coauthors to register for an ORCID iD and use it as well. Please encourage your coauthors to verify their ORCID iD within the submission system before final acceptance, as unverified ORCID iDs will not appear in the published article. *Only*  the individual author can complete the verification step; PLOS staff *cannot*  verify ORCID iDs on behalf of authors.

We look forward to receiving your revised manuscript.

Kind regards,

Ricardo Ney Oliveira Cobucci, Ph.D

Academic Editor

PLOS One

Journal Requirements:

1. If the reviewer comments include a recommendation to cite specific previously published works, please review and evaluate these publications to determine whether they are relevant and should be cited. There is no requirement to cite these works unless the editor has indicated otherwise.

2. Please review your reference list to ensure that it is complete and correct. If you have cited papers that have been retracted, please include the rationale for doing so in the manuscript text, or remove these references and replace them with relevant current references. Any changes to the reference list should be mentioned in the rebuttal letter that accompanies your revised manuscript. If you need to cite a retracted article, indicate the article’s retracted status in the References list and also include a citation and full reference for the retraction notice.

[Note: HTML markup is below. Please do not edit.]

Reviewers' comments:

Reviewer's Responses to Questions

**Comments to the Author**

Reviewer #1: All comments have been addressed

Reviewer #2: (No Response)

Reviewer #5: All comments have been addressed

**********

2. Is the manuscript technically sound, and do the data support the conclusions?

Reviewer #1: Yes

Reviewer #2: Yes

Reviewer #5: Yes

**********

3. Has the statistical analysis been performed appropriately and rigorously?

Reviewer #1: Yes

Reviewer #2: Yes

Reviewer #5: Yes

**********

4. Have the authors made all data underlying the findings in their manuscript fully available?

The PLOS Data policy

Reviewer #1: Yes

Reviewer #2: Yes

Reviewer #5: Yes

**********

5. Is the manuscript presented in an intelligible fashion and written in standard English?

Reviewer #1: Yes

Reviewer #2: Yes

Reviewer #5: No

**********

Reviewer #1: The revision has been done well; however, one issue still requires clarification: Were the 62.2% of women excluded from PCOS diagnosis or were their PCOM-status reclassified (phenotype changed)? Authors state they have changed this, but still in the Discussion (chapter starting with "A study was conducted...") it is said "In our study, 62.2% of women were excluded from PCOS diagnosis as per new cutoff." Please make changes everywhere in the text.

Please also add a table showing phenotype shifts based on the results. How many women would really fall out from PCOS group and how many would be reclassified into different phenotypes.

It is good that studies are conducted in populations other than Caucasians, so this article is of importance.

Reviewer #2: I think the title does not adequately describe the topic being studied. This will decrease interest in reading the article.

The citations are not in order and some articles listed in the references don't appear to be cited in the article.

Reviewer #5: ◦ This article is quite clinically and scientifically relevant; however, it does need some revision for clarity. The revised version appears to address the important PCOS question: whether raising the AFC threshold from 12 to 20 may inadvertently exclude a significant number of women from the diagnosis. The study results suggest that women with AFC 12 - 19 still have both metabolic and endocrine features similar to women with confirmed PCOS, so technically, the revised cutoff may lead to underdiagnosis. The introduction and objectives are clearly stated, and the methodology is good, with a good sample size, standardized diagnostic definitions, and statistical analysis. The discussion also connects the findings well to previous studies and highlights the clinical importance. Unfortunately, the main weaknesses of the study are the misleading title, some grammar and formatting problems, long repetitive sentences, and some overinterpretation of results.

◦ Overall, the study has good scientific value and can be recommended for publication after revision. The review recommends improving the title, formatting the writing, organizing the results more clearly, softening a few conclusions, and strengthening the limitations section.

**********

what does this mean?). If published, this will include your full peer review and any attached files.

If you choose “no”, your identity will remain anonymous but your review may still be made public.

**Do you want your identity to be public for this peer review?**  For information about this choice, including consent withdrawal, please see our Privacy Policy

Reviewer #1: No

Reviewer #2: No

Reviewer #5: **Yes:**  Ekwuazi Kingsley Emeka

**********

[NOTE: If reviewer comments were submitted as an attachment file, they will be attached to this email and accessible via the submission site. Please log into your account, locate the manuscript record, and check for the action link "View Attachments". If this link does not appear, there are no attachment files.]

To ensure your figures meet our technical requirements, please review our figure guidelines: https://journals.plos.org/plosone/s/figures

You may also use PLOS’s free figure tool, NAAS, to help you prepare publication quality figures: https://journals.plos.org/plosone/s/figures#loc-tools-for-figure-preparation.

NAAS will assess whether your figures meet our technical requirements by comparing each figure against our figure specifications.

# Author comment: Author response to Decision Letter 2

**DOI:** 10.1371/journal.pone.0358554.r004

20 May 2026

We sincerely thank the Editor and the Reviewers for their careful evaluation of our manuscript and for providing insightful and constructive comments. We have revised the manuscript thoroughly in response to these suggestions, and we believe that these changes have significantly improved the clarity and quality of our work.

All comments have been addressed point-by-point. For ease of review, the reviewers’ comments are presented in italic, followed by our responses. Changes made in the manuscript have been highlighted. Response to all the comments has been uploaded as a separate file named as response to reviewer.

Attachment Submitted filename: Response to reviewers.docx

# Peer review: Decision Letter 2

**DOI:** 10.1371/journal.pone.0358554.r005

**Authors:** Ricardo Cobucci

2 Jun 2026

Dear Dr. Malhotra,

Thank you for submitting your manuscript to PLOS ONE. After careful consideration, we feel that it has merit but does not fully meet PLOS ONE’s publication criteria as it currently stands. Therefore, we invite you to submit a revised version of the manuscript that addresses the points raised during the review process.

Please submit your revised manuscript by Jul 17 2026 11:59PM. If you will need more time than this to complete your revisions, please reply to this message or contact the journal office at plosone@plos.org. When you're ready to submit your revision, log on to https://www.editorialmanager.com/pone/ and select the 'Submissions Needing Revision' folder to locate your manuscript file.

- A letter that responds to each point raised by the academic editor and reviewer(s). You should upload this letter as a separate file labeled 'Response to Reviewers'.
- A marked-up copy of your manuscript that highlights changes made to the original version. You should upload this as a separate file labeled 'Revised Manuscript with Track Changes'.
- An unmarked version of your revised paper without tracked changes. You should upload this as a separate file labeled 'Manuscript'.

If you would like to make changes to your financial disclosure, please include your updated statement in your cover letter. Guidelines for resubmitting your figure files are available below the reviewer comments at the end of this letter.

If applicable, we recommend that you deposit your laboratory protocols in protocols.io to enhance the reproducibility of your results. Protocols.io assigns your protocol its own identifier (DOI) so that it can be cited independently in the future. For instructions see: https://journals.plos.org/plosone/s/submission-guidelines#loc-laboratory-protocols. Additionally, PLOS ONE offers an option for publishing peer-reviewed Lab Protocol articles, which describe protocols hosted on protocols.io. Read more information on sharing protocols at https://plos.org/protocols?utm_medium=editorial-email&utm_source=authorletters&utm_campaign=protocols.

As the corresponding author, your ORCID iD is verified in the submission system and will appear in the published article. PLOS supports the use of ORCID, and we encourage all coauthors to register for an ORCID iD and use it as well. Please encourage your coauthors to verify their ORCID iD within the submission system before final acceptance, as unverified ORCID iDs will not appear in the published article. *Only*  the individual author can complete the verification step; PLOS staff *cannot*  verify ORCID iDs on behalf of authors.

We look forward to receiving your revised manuscript.

Kind regards,

Ricardo Ney Oliveira Cobucci, Ph.D

Academic Editor

PLOS One

Journal Requirements:

1. If the reviewer comments include a recommendation to cite specific previously published works, please review and evaluate these publications to determine whether they are relevant and should be cited. There is no requirement to cite these works unless the editor has indicated otherwise.

2. Please review your reference list to ensure that it is complete and correct. If you have cited papers that have been retracted, please include the rationale for doing so in the manuscript text, or remove these references and replace them with relevant current references. Any changes to the reference list should be mentioned in the rebuttal letter that accompanies your revised manuscript. If you need to cite a retracted article, indicate the article’s retracted status in the References list and also include a citation and full reference for the retraction notice.

[Note: HTML markup is below. Please do not edit.]

Reviewer's Responses to Questions

**Comments to the Author**

Reviewer #1: All comments have been addressed

Reviewer #5: All comments have been addressed

**********

2. Is the manuscript technically sound, and do the data support the conclusions?

Reviewer #1: Yes

Reviewer #5: Yes

**********

3. Has the statistical analysis been performed appropriately and rigorously?

Reviewer #1: Yes

Reviewer #5: I Don't Know

**********

4. Have the authors made all data underlying the findings in their manuscript fully available?

The PLOS Data policy

Reviewer #1: Yes

Reviewer #5: Yes

**********

5. Is the manuscript presented in an intelligible fashion and written in standard English?

Reviewer #1: Yes

Reviewer #5: Yes

**********

Reviewer #1: Please spell out the abbreviation AMH when first mentioned.

Still, the authors claim “Practically, the data indicate that using ≥20 follicles as a diagnostic cut-point would reclassify 62% of symptomatic women as “non-PCOS” despite AMH levels consistent with ovarian hyperactivity.”

Please make a table, that indicates:

a.) Phenotypes with the cut-off of 12

b.) Phenotypes with the cut-off of 20 and how many women would lose PMOS diagnosis

Then you can answer the question of how many women would be left undiagnosed if the cut-off of 12 is utilized.

Reviewer #5: The manuscript has undergone a significant improvement after revision, particularly in acknowledging its limitations and clarifying the interpretation of AMH. The discussion appropriately refrains from asserting that AMH serves as a replacement diagnostic criterion.

The manuscript alternates between “cross-sectional” and “retrospective observational”, which are unfortunately, not interchangeable. The study should be best described as a retrospective cross-sectional observational study. I suggest that the researchers ensure consistency in their terminology.

**********

what does this mean?). If published, this will include your full peer review and any attached files.

If you choose “no”, your identity will remain anonymous but your review may still be made public.

**Do you want your identity to be public for this peer review?**  For information about this choice, including consent withdrawal, please see our Privacy Policy

Reviewer #1: No

Reviewer #5: **Yes:**  Ekwuazi Kingsley Emeka

**********

[NOTE: If reviewer comments were submitted as an attachment file, they will be attached to this email and accessible via the submission site. Please log into your account, locate the manuscript record, and check for the action link "View Attachments". If this link does not appear, there are no attachment files.]

To ensure your figures meet our technical requirements, please review our figure guidelines: https://journals.plos.org/plosone/s/figures

You may also use PLOS’s free figure tool, NAAS, to help you prepare publication quality figures: https://journals.plos.org/plosone/s/figures#loc-tools-for-figure-preparation.

NAAS will assess whether your figures meet our technical requirements by comparing each figure against our figure specifications.

# Author comment: Author response to Decision Letter 3

**DOI:** 10.1371/journal.pone.0358554.r006

4 Aug 2026

Reviewer comment:

Reviewer #1

Please spell out the abbreviation AMH when first mentioned.

Still, the authors claim: “Practically, the data indicate that using ≥20 follicles as a diagnostic cut-point would reclassify 62% of symptomatic women as ‘non-PCOS’ despite AMH levels consistent with ovarian hyperactivity.”

Please make a table that indicates:

a) Phenotypes with the cut-off of 12

b) Phenotypes with the cut-off of 20 and how many women would lose PCOS diagnosis

Then you can answer the question of how many women would be left undiagnosed if the cut-off of 12 is utilized.

Response:

We thank the reviewer for this important comment. We have now spelled out anti-Müllerian hormone (AMH) at its first mention in the manuscript.

We agree that our previous statement that increasing the follicle-count threshold to ≥20 would reclassify approximately 62% of symptomatic women as “non-PCOS” was imprecise. Loss of the polycystic ovarian morphology criterion does not necessarily imply loss of the PCOS diagnosis, because women fulfilling both the oligo-anovulation and hyperandrogenism criteria continue to meet the Rotterdam diagnostic criteria.

We therefore performed a phenotype-wise reclassification using an antral follicle count threshold of ≥12 and subsequently ≥20 follicles. The revised phenotype distribution and reclassification are presented in the following table.

Original phenotype using AFC ≥12 n (%) Classification after applying AFC ≥20 n (%)

A: OA + HA + PCOM 190 (56.7) A: OA + HA + PCOM 62 (18.5)

B: OA + HA 128 (38.2)

B: OA + HA 13 (3.9) B: OA + HA 13 (3.9)

C: HA + PCOM 104 (31.0) C: HA + PCOM 32 (9.6)

Hyperandrogenism only 72 (21.5)

D: OA + PCOM 28 (8.4) Oligo-anovulation only 28 (8.4)

No longer fulfilling the Rotterdam criteria — 100 (29.9)

Among the 190 women with phenotype A at the AFC ≥12 threshold, 128 had an AFC of 12–19 and therefore lost the PCOM criterion when the threshold was increased to ≥20. However, these women continued to fulfil both the oligo-anovulation and hyperandrogenism criteria and were reclassified as phenotype B. They therefore retained the diagnosis of PCOS.

Among the 104 women with phenotype C, 72 lost the PCOM criterion and were reclassified as having isolated hyperandrogenism. Similarly, all 28 women with phenotype D lost the PCOM criterion and were reclassified as having isolated oligo-anovulation. These women no longer fulfilled at least two of the three Rotterdam criteria.

Consequently, 100 of the 335 women originally diagnosed with PCOS using AFC ≥12, corresponding to 29.9%, would no longer fulfil the Rotterdam criteria when the AFC threshold was increased to ≥20. The remaining 235 women (70.1%) would retain the diagnosis of PCOS.

We have therefore replaced the previous statement with the following in the Results section:

“Increasing the AFC threshold for polycystic ovarian morphology from ≥12 to ≥20 follicles resulted in loss of the PCOM criterion in 228 of 335 women (68.1%). Of these, 128 women originally classified as phenotype A continued to fulfil the oligo-anovulation and hyperandrogenism criteria and were reclassified as phenotype B, while 62 remained phenotype A. All 13 women with phenotype B remained unchanged. Among women with phenotype C, 72 lost the PCOM criterion and were reclassified as having isolated hyperandrogenism, while 32 remained phenotype C. All 28 women with phenotype D were reclassified as having isolated oligo-anovulation. Overall, 100 of 335 women (29.9%) no longer fulfilled the Rotterdam diagnostic criteria for PCOS after applying the AFC ≥20 threshold.”

The changes made in the discussion as well

“From a practical standpoint, increasing the diagnostic threshold for polycystic ovarian morphology from ≥12 to ≥20 follicles would result in 29.9% of women no longer meeting the Rotterdam criteria for PCOS. Given that many of these women continued to demonstrate biochemical evidence of ovarian hyperactivity, including elevated AMH levels, these findings raise concerns that the revised threshold may reduce diagnostic sensitivity in symptomatic women with less marked follicular excess.”

Reviewer #5:

The manuscript has undergone a significant improvement after revision, particularly in acknowledging its limitations and clarifying the interpretation of AMH. The discussion appropriately refrains from asserting that AMH serves as a replacement diagnostic criterion.

The manuscript alternates between “cross-sectional” and “retrospective observational”, which are unfortunately not interchangeable. The study should be best described as a retrospective cross-sectional observational study. I suggest that the researchers ensure consistency in their terminology.

Response:

We thank the reviewer for the positive comments on our revisions and for this helpful suggestion. We agree that the terminology should be consistent throughout the manuscript. Accordingly, we have revised the manuscript to describe the study uniformly as a retrospective cross-sectional observational study in the Abstract, Methods, and elsewhere in the manuscript, replacing instances where only "cross-sectional" or "retrospective observational" had been used. This terminology more accurately reflects the study design and has been applied consistently throughout the revised manuscript.

Attachment Submitted filename: Response to reviewers 1.docx

# Peer review: Decision Letter 3

**DOI:** 10.1371/journal.pone.0358554.r007

**Authors:** Ricardo Cobucci

3 Sep 2026

Impact of Revised Antral Follicle Count Threshold on PCOS Diagnosis: A Comparative Clinical Study

PONE-D-25-60592R3

Dear Dr. Malhotra,

We’re pleased to inform you that your manuscript has been judged scientifically suitable for publication and will be formally accepted for publication once it meets all outstanding technical requirements.

Within one week, you’ll receive an e-mail detailing the required amendments. When these have been addressed, you’ll receive a formal acceptance letter and your manuscript will be scheduled for publication.

An invoice will be generated when your article is formally accepted. Please note, if your institution has a publishing partnership with PLOS and your article meets the relevant criteria, all or part of your publication costs will be covered. Please make sure your user information is up-to-date by logging into Editorial Manager at Editorial Manager® and clicking the ‘Update My Information' link at the top of the page. For questions related to billing, please contact billing support.

If your institution or institutions have a press office, please notify them about your upcoming paper to help maximize its impact. If they’ll be preparing press materials, please inform our press team as soon as possible -- no later than 48 hours after receiving the formal acceptance. Your manuscript will remain under strict press embargo until 2 pm Eastern Time on the date of publication. For more information, please contact onepress@plos.org.

Kind regards,

Ricardo Ney Oliveira Cobucci, Ph.D

Academic Editor

PLOS One

Additional Editor Comments (optional):

Reviewers' comments:

Reviewer's Responses to Questions

**Comments to the Author**

Reviewer #5: All comments have been addressed

**********

2. Is the manuscript technically sound, and do the data support the conclusions?

Reviewer #5: Yes

**********

3. Has the statistical analysis been performed appropriately and rigorously?

Reviewer #5: Yes

**********

4. Have the authors made all data underlying the findings in their manuscript fully available?

The PLOS Data policy

Reviewer #5: Yes

**********

5. Is the manuscript presented in an intelligible fashion and written in standard English?

Reviewer #5: Yes

**********

Reviewer #5: Overall, I would rate the manuscript as potentially publishable with some additional revisions, rather than requiring a fundamental redesign. The study question, dataset, phenotype analysis, and comparison of the 12–19 group with ≥20 are the strongest components. The paper will become much stronger if it presents the ≥20 threshold as a change in PCOM classification with downstream effects on PCOS phenotyping, rather than as a direct replacement diagnostic test for PCOS.

**********

what does this mean?). If published, this will include your full peer review and any attached files.

If you choose “no”, your identity will remain anonymous but your review may still be made public.

**Do you want your identity to be public for this peer review?**  For information about this choice, including consent withdrawal, please see our Privacy Policy

Reviewer #5: **Yes:**  Ekwuazi Kingsley Emeka

**********

# Editor report: Acceptance letter

**DOI:** 10.1371/journal.pone.0358554.r008

**Authors:** Ricardo Cobucci

PONE-D-25-60592R3

PLOS One

Dear Dr. Malhotra,

I'm pleased to inform you that your manuscript has been deemed suitable for publication in PLOS One. Congratulations! Your manuscript is now being handed over to our production team.

At this stage, our production department will prepare your paper for publication. This includes ensuring the following:

* All references, tables, and figures are properly cited

* All relevant supporting information is included in the manuscript submission,

* There are no issues that prevent the paper from being properly typeset

You will receive further instructions from the production team, including instructions on how to review your proof when it is ready. Please keep in mind that we are working through a large volume of accepted articles, so please give us a few days to review your paper and let you know the next and final steps.

Lastly, if your institution or institutions have a press office, please let them know about your upcoming paper now to help maximize its impact. If they'll be preparing press materials, please inform our press team within the next 48 hours. Your manuscript will remain under strict press embargo until 2 pm Eastern Time on the date of publication. For more information, please contact onepress@plos.org.

You will receive an invoice from PLOS for your publication fee after your manuscript has reached the completed accept phase. If you receive an email requesting payment before acceptance or for any other service, this may be a phishing scheme. Learn how to identify phishing emails and protect your accounts at https://explore.plos.org/phishing.

If we can help with anything else, please email us at customercare@plos.org.

Thank you for submitting your work to PLOS One and supporting open access.

Kind regards,

PLOS One Editorial Office Staff

on behalf of

PROFESSOR Ricardo Ney Oliveira Cobucci

Academic Editor

PLOS One
